# Large‐Area 2D Metasurface‐Based Triboelectric E‐Skin Arrays: Contact & Proximity Tactile Mapping with Broadband Acoustic Readouts

**DOI:** 10.1002/adma.202521525

**Published:** 2026-02-11

**Authors:** Injamamul Arief, Swagato Sarkar, Anik Kumar Ghosh, Osvalds Verners, Kamal Kumar Meena, Su‐Hyeong Lee, Soosang Chae, Tobias A. F. König, Beate Krause, Andreas Fery, Mehmet Sait Özer, Anindya Nag, Amit Das

**Affiliations:** ^1^ Department of Elastomers Division Polymer Materials Engineering Leibniz Institute of Polymer Research Dresden Hohe Str. 6 Dresden Germany; ^2^ Department of Functional Colloidal Materials Division Physical Chemistry and Physics of Polymers Leibniz Institute of Polymer Research Dresden Hohe Str. 6 Dresden Germany; ^3^ Institute of Materials and Surface Engineering Faculty of Natural Sciences and Technologies Riga Technical University Riga Latvia; ^4^ School of Energy Materials and Chemical Engineering Korea University of Technology and Education Cheonan South Korea; ^5^ Faculty of Chemistry and Food Chemistry Technische Universität Dresden Dresden Germany; ^6^ Dresden Center for Intelligent Materials (DCIM) Technische Universität Dresden Dresden Germany; ^7^ Department of Sustainable Polymer Structures Division Macromolecular Chemistry Leibniz Institute of Polymer Research Dresden Dresden Germany; ^8^ Chair For Physical Chemistry of Polymeric Materials Technische Universität Dresden Dresden Germany; ^9^ Faculty of Electrical and Computer Engineering Technische Universität Dresden Dresden Germany; ^10^ Centre For Tactile Internet with Human‐in‐the‐Loop (CeTI) Technische Universität Dresden Dresden Germany

**Keywords:** acoustic sensing, dielectric metasurfaces, human‐machine interaction, proximity recognition, triboelectric nanogenerators

## Abstract

Recent advances in electromechanically coupled, self‐powered, flexible transducer‐enabled electronic skins are predominantly driven by the capacitive triboelectric nanogenerators (TENGs), which operate intrinsically as multifunctional sensor‐cum‐energy harvester. The resulting TENG's operability in cutting‐edge wearable technologies can be significantly augmented by introducing 2D dielectric metasurfaces, which optimize functionality through enhanced electromechanical coupling. Here, we introduce a 2D metasurface‐TENG e‐skin that unifies tactile (contact and inductive) and acoustic sensing in a single ultrathin platform. Large‐area nanocone (NC) metasurfaces are engineered on 100 µm polydimethoxysilane (PDMS) films via laser‐interference lithography (LIL) and soft molding, which boosts triboelectric charge density and provides optical diffraction cues for strain monitoring. Integrated into a 3 × 3 array, the device delivers real‐time tactile pressure imaging with low crosstalk and non‐contact proximity detection. The NC‐TENG patch also functions as a self‐powered acoustic sensor, in which the sound pressure level (SPL) and frequency response are quantified in both spatial and spectral domains over a broad frequency range (∼50–6400 Hz). Compared to pristine PDMS, the metasurface enhances open‐circuit voltage by ≈46% under identical loading and sustains stable electrical output. By coupling electromechanical and electro‐acoustic transductions with metasurface optics, this work advances multimodal, arrayed e‐skins for next‐generation human‐machine interfaces and wearable sensing.

## Graphical abstract

Metasurface‐engineered NC‐TENG arrays integrate tactile pressure mapping, non‐contact gesture sensing, and acoustic signal readouts in one ultrathin module, and outperforms pristine PDMS in terms of electrical output and real‐time spatial mapping for next‐gen wearables.

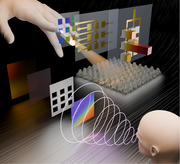

## 1 Introduction

The current interest in ultrathin, stretchable‐flexible personal electronics has already reached its pinnacle, and technologies of soft electronics & associated devices have evolved remarkably over the last decades. As a matter of fact, multifunctionality and wearability have become the newest technological trends. Recent global multidisciplinary research efforts have generated numerous innovations in wearable devices, artificial skin, soft robotics, and deformable electronics [1, 2, 3]. It has to be noted that human‐machine interaction is an important aspect of wearable technology, as it allows the exchange of information between people and electronics. Tactile devices capable of transducing physical touch into electrical signals are regarded as one of the most important HMI devices. Wide‐area pressure sensor arrays are based on transistor switches and offer several advantages, such as reduced power consumption and crosstalk among the array points [4, 5, 6, 7]. However, tactile sensors are mainly based on the principles of surface acoustic wave, infrared, capacitive, and resistive sensing [8]. These surface acoustic wave‐based sensors are often subjected to interference from the surroundings, whereas the infrared sensors rely heavily on complex light‐emitting diode beams and photodetector arrays [9, 10]. The resistive sensors, on the other hand, demonstrate reduced sensitivity and higher detection limits at very low pressures [11, 12]. Out of all, capacitive sensors are most widely employed in tactile devices and electronic skins and are considerably sensitive to milder touch recognition [13]. However, the output signal for resistive and capacitive sensors changes almost immediately when the external contact pressure disappears, complicating continuous monitoring and imaging using serial data readings. Additionally, only touch and sliding gestures can be recognized, which limits their usage in other modes of gesture recognition, such as non‐contact sensing [14]. Furthermore, optical‐derived pressure sensor matrix based on piezophototronic effect showed high resolution, albeit with low pressure sensitivity [15].

Recently, triboelectric nanogenerators (TENGs) have gained attention as in situ power sources and active sensors that convert mechanical stimuli into electrical signals [16, 17, 18]. TENG‐based tactile sensors perform by coupling contact electrification and electrostatic induction to produce strong output voltages from gentle touches, enabling highly sensitive pressure detection without any external power [16, 17, 18, 19, 20, 21, 22, 23]. They offer advantages of low cost, light weight, and mechanical flexibility, making them ideal for wearable applications. Importantly, TENG arrays have demonstrated real‐time pressure mapping with high spatial resolution in electronic skins by using either single‐electrode or paired‐electrode configurations that localize touch inputs [19, 20, 24, 25, 26, 27, 28]. In addition to contact mode sensing, TENG devices can operate in a non‐contact mode in which a grounded TENG electrode can detect the approach or motion of a charged object (like a human finger) without physical contact [14]. This capability has been employed to recognize diverse hand gestures in touch‐free screens, overcoming the limitations of capacitive sensors [14, 19].

As defined previously, user‐device interaction is a crucial parameter in any wearable, interactive technology. Among the five human senses, most current developments are inevitably focused on sight and touch [29]. However, an optimal wearable IoT device should incorporate other senses to enable a richer interactive experience in a truly multimodal interface, tailored to specific applications. Acoustic transduction has been highly regarded as a subject of interest in the low‐power energy transfer segment [29, 30, 31]. This is reflected in voice command‐activated devices in consumer electronics, speech converters, and voice recognition systems. Several studies in triboelectric acoustic sensors (TAS) have shown that TENG‐based membranes can serve as self‐powered microphones, converting sound‐induced vibrations into electrical signals across a wide range of frequencies [29, 30, 31, 32]. As most TAS operate in membrane‐based diaphragm design, it is highly efficient in harnessing acoustic signals in the audible ranges (hundreds to thousands of Hz). For example, single‐electrode TENG diaphragms have been used to detect voice and environmental sounds, demonstrating the feasibility of triboelectric sensors for acoustic monitoring [33]. As researchers attempt to combine multiple functionalities into a single sensor, multimodal sensors are a highly attractive platform for detecting more than one stimulus [34]. This paper exemplifies the first true multimodality in a TENG‐based interface combining contact, noncontact gesture recognition, and acoustic sensing into one.

Textures and patterned microstructures on a triboelectric surface have been explored significantly as feasible means to improve the contact electrification (CE) performance of a TENG. The enhanced CE effect eventually improves the output signal and hence, the signal‐to‐noise ratio (SNR) [35, 36]. Beyond increased surface area, periodic nanopatterning toward metasurface development can also induce optical effects that play a vital role in characterizing and confirming the functionalities of TENG interfaces. Generally, for a dielectric metasurface (considering a 1D periodic grating as the simplest case), when the periodicity of the nanostructures is comparable to the wavelength of interacting light, interesting phenomena like Mie scattering and photonic diffractions come into play [37, 38]. These phenomena can be exploited to modulate the interaction between light and matter, enabling features like selective filtering, wave guiding, and enhanced light absorption [39]. In the context of TENG interfaces, these optical effects can be utilized to develop integrated functionalities such as touch and gesture recognition alongside optical sensing capabilities. However, for a systematic evaluation, it is essential to characterize an ordered system with homogeneity in terms of a large surface area to predict a consistent response across the metasurface, thereby further increasing the signal‐to‐noise ratio [40]. Since the response from a small portion of the surface then can be extended or considered for the entire metasurface, numerical simulations with periodic boundary conditions for such ordered system can save considerable amount of simulation time. Further, such periodic surfaces exhibit Rayleigh anomalies, where specific wavelengths of light experience an abrupt change in transmittance due to the periodic arrangement of features [39, 40]. The key point is that the spectral position of these anomalies is directly related to the spacing between the repeated nanostructures [41]. This principle can be utilized to develop highly sensitive and versatile strain sensors based on TENGs [42]. A recent study demonstrated a gratings‐based TENG sensor exhibiting a high gauge factor (over 1000) and excellent stretchability, making it ideal for strain‐sensing applications [42]. Furthermore, the flexibility of elastomeric polymers (e.g. polydimethylsiloxane, PDMS) can be combined with nanopatterning to create stretchable and wearable TENG interfaces [43, 44].

**FIGURE 1:**
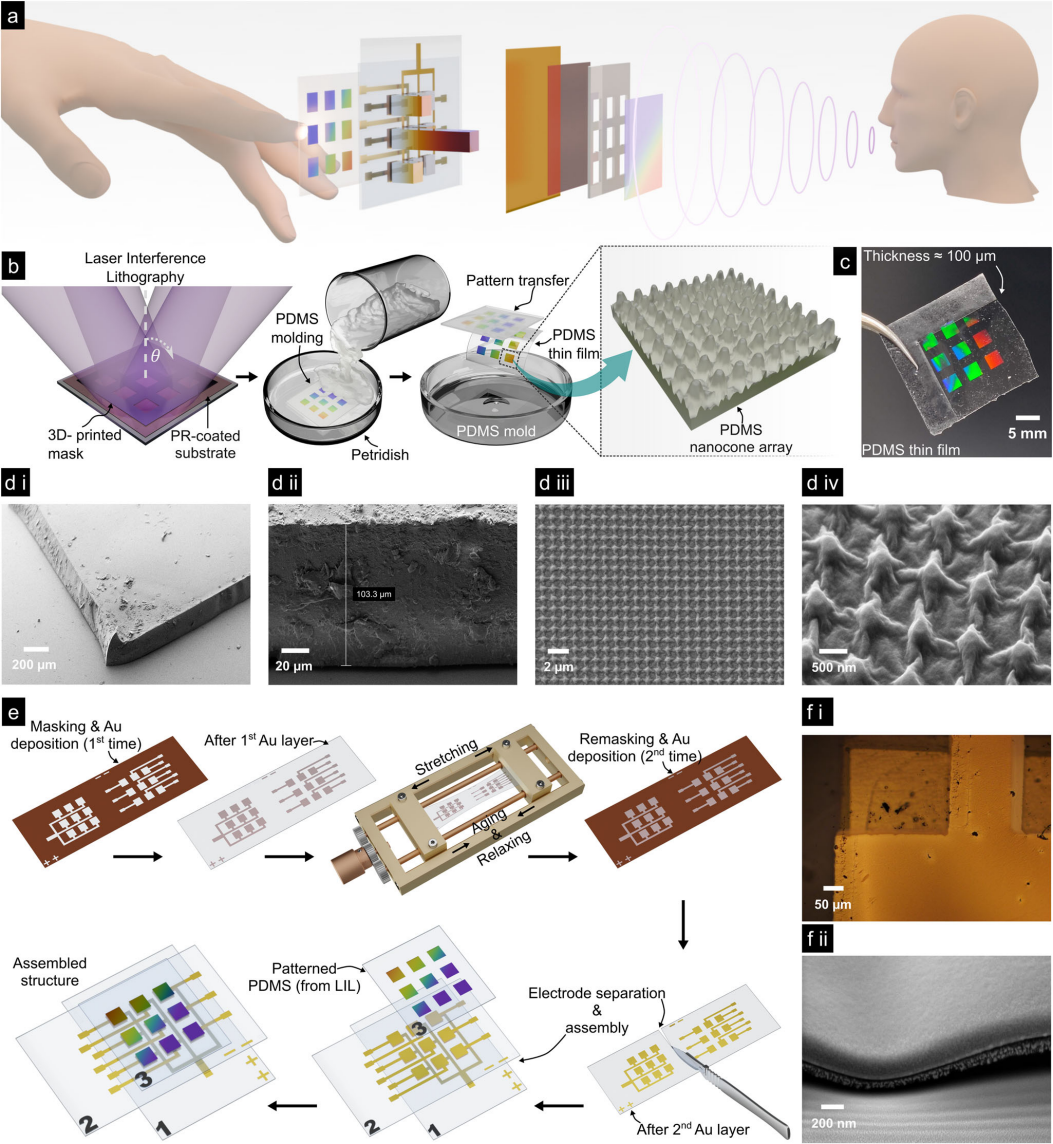
(a) Schematic overview showing the triboelectric and acoustic applications of the nanocone 2D‐array based metasurface. (b) LIL with an additional 3D printed 3 × 3 photomask. 2D periodic hole array in the photoresist layer, later transferred to a PDMS thin film through a soft‐molding process. (c) Photographic image of the patterned PDMS thin film with 100‐micron thickness. (d, i‐ii) SEM image confirming the PDMS thin sheet of ∼100‐micron thickness. (iii) SEM image (top view) of the TENG metasurface showing uniformity over a large area. (iv) Zoomed inset shows 3D view of the patterned region showing the PDMS nanocone (NC) array. (e) Fabrication of stretchable gold thin film electrode utilizing metal‐elastomer‐metal nanostructure. (f, i‐ii) Top‐view optical microscopy image and cross‐sectional FIB image of the deposited metal‐elastomer‐metal electrode.

The 2D periodic patterning requires soft lithography, which involves creating an inverse design from a 2D patterned photoresist master. The readily available commercial masters offer convenience; however, they limit scalability, choice, and reconfigurability. Further, since electron beam lithography is generally used to create these custom masters with predefined aspect ratios, its application is restricted to small patterning areas. In this regard, laser interference lithography (LIL) emerges as the optimal solution, balancing the need for large‐area patterning with the ability to achieve high aspect ratio structures cost‐effectively [45, 46]. We have previously employed LIL to fabricate a variety of 1D and 2D templates by generating PDMS replicas, which were subsequently utilized for nanophotonic applications based on template‐assisted self‐assembly of plasmonic [47, 48] and semiconductor nanocrystals/quantum dots [49, 50]. However, the thickness of these replicas (avg. 1–2 mm) renders them unsuitable for the current application. In this work, a 325 nm He‐Cd laser LIL setup is used to create a 2D array of nano‐holes in a photoresist layer, with a custom 3 × 3 photomask to delineate discrete sensing pixels. We then transfer this pattern into a thin (100 µm) silicone film via soft lithographic molding, yielding a continuous PDMS nanocone array that is flexible and robust. This nanocone TENG (NC‐TENG) metasurface forms the active layer of our sensor. By integrating it into a 3 × 3 matrix with an underlying stretchable electrode architecture, we realize a self‐powered e‐skin capable of multi‐point tactile sensing, proximity detection, and acoustic response. To the best of our knowledge, this work represents the first demonstration of a tri‐modal triboelectric sensor array that seamlessly merges contact, non‐contact, and acoustic sensing functionalities as shown schematically Figure 1a.

## 2 Results and Discussion

### 2.1 Fabrication of 2D Metasurface‐Based Triboelectric Nanogenerators

The fabrication of the TENG metasurface begins with LIL (Figure 1b), which defines a 2D periodic hole array within a photoresist layer (see Experimental Section). This patterning step ensures precise large‐area periodicity, a prerequisite for optimizing triboelectric energy conversion. In the present case, a spatially separated patterned region is defined on the photoresist master using a masking technique. This region comprises 3 × 3 blocks (nine blocks in total, each measuring 2 mm × 2 mm with an interspacing of 1 mm), which is essential for the subsequent PDMS molding process. Figure S1 of the Supporting Information text describes the details of the LIL setup. Depending on the excitation configuration, each patterned block exhibits a 2D periodic hole array generated through a dual‐exposure LIL process, in which the photoresist‐coated glass substrate is rotated by 90° between two successive exposures (Figure S1a). To ensure precise alignment of the patterning regions during the in‐plane azimuthal rotation, a 3 × 3 mask is affixed to the coated substrate, allowing both the mask and the substrate to rotate synchronously. In this manner, an initial exposure produced 1D periodic lines in each of the nine blocks, followed by a second orthogonal exposure at the same locations to define the 2D lattice. After development, the overexposed photoresist regions are removed, yielding well‐ordered 2D hole arrays within each block (Figure S1b(i, ii)). Figure S2 in the Supporting Information presents the corresponding AFM images of the resulting structures, illustrating variations in periodicity achieved by tuning the interference angles within the LIL set‐up, ranging from 500 to 1000 nm. After considering the depth profile and the aspect ratio of the holes, we have proceeded with the 1000 nm periodicity to replicate well‐defined nanocones following the pattern transfer.

After confirming with the metasurface design, a PDMS prepolymer mixture consisting of an elastomer and curing agent in a 10:1 ratio is gently poured into a plastic Petri dish containing the patterned photoresist master. Notably, the master is placed face‐down, allowing the PDMS to conform precisely to the nanostructured surface during desiccation, thereby ensuring accurate replication of the nanocone features through the soft‐molding process. In contrast to previously reported configurations [48, 49, 50], where the master faced upward and the poured volume governed the PDMS thickness, the inverted orientation employed here enabled the formation of uniform PDMS films with micron‐scale thickness control. Prolonged degassing in a desiccator further facilitated the infiltration of the liquid PDMS between the patterned master and the Petri dish surface, ensuring complete feature replication and minimizing trapped air pockets. Further, as the PDMS cures at a controlled temperature of 60°C for the next 3 h, it conforms to the contours of the photoresist pattern, thus capturing the intricate details of the microstructured surface. Once cured, the solidified PDMS, along with the photoresist master, is gently cut off from the Petri dish. After removing the thicker PDMS layer from the back of the glass substrate of the photoresist master, the thin PDMS layer from the patterned surface is gently peeled off using precision tweezers. This peeling process reveals the successful transfer of nanocone structures from the master to the thin PDMS film. The photographic image in Figure 1c shows the PDMS film with a 3 × 3 patterned region. The vibrant coloration marks the patterned areas due to the optical properties of the nanostructures, providing a sharp contrast between the accurately patterned and non‐patterned regions. Next, scanning electron microscopy (SEM) is utilized to confirm the microscale details of the fabricated thin‐film PDMS metasurface. A consistent thickness across the fabricated sample is essential for uniform electrical performance in triboelectric applications. Figure 1d(i, ii) shows the precise measurement of the film's thickness ∼100 µm and provide a topographical view of the metasurface film. Moreover, the SEM image in Figure 1d(iii) showcases an expanded view of the nanocone pattern with a periodicity of 1 µm, highlighting its uniformity across a large area, which is vital for scaling up the technology for commercial applications (Figure S3). The detailed 3D view of the nanocones further illustrates the sharp and well‐defined features achieved through this accurate fabrication process (Figure 1d(iv)). The SEM images of the 2D‐metasurface illustrating large‐scale and high‐resolution nanocone geometries are provided in Figure S4a,b (high and low resolution, respectively) of the Supporting Information.

In order to ensure the stable electrical performance of the device under repeated mechanical deformation, we employ a stretchable electrode based on a metal‐elastomer‐metal (M‐E‐M) nanostructure [51], following the protocol shown in Figure 1e. First, a thin (100 µm) PDMS film is prepared on a supporting substrate, and a first Au layer is deposited through a shadow mask to define the electrode pads and interconnects (Figure 1e, Masking & Au deposition (first time)). The patterned PDMS/Au sample is then mounted on a stretching device, uniaxially stretched (80%), and aged under strain at high vacuum (≈ 6.0 × 10^−6^ mbar) for 8 h [51]. During this step, low molecular weight PDMS chains migrate out of the bulk and form a thin, compliant barrier layer at the metal‐elastomer interface, yielding a soft tunneling layer with the Au film stack. After the sample is relaxed to its original length, the same mask is realigned and a second thin Au film is deposited (re‐masking & Au deposition, second time), thereby forming a sandwiched Au‐PDMS‐Au nanostructure on the elastomer substrate. The optical micrographs of the Au electrode patterns and connectors are shown in Figure S5. Two electrode patterns are fabricated simultaneously on the same PDMS sheet; they are first mechanically separated and then assembled in a face‐to‐face configuration to form the final pair of stretchable electrodes, as shown in the second panel from the bottom left of Figure 1e. Subsequently, the topmost patterned PDMS layer is treated with air plasma and bonded onto this face‐to‐face electrode pair, yielding the complete device structure illustrated in the extreme bottom‐left panel of Figure 1e. For clearer visualization of this stacked architecture, the individual layers are labeled 1–3 from bottom to top, and the (++) and (–) markers are used to help the reader track how the matching layers are aligned on top of one another in Figure S6 of the Supporting Information.

As shown in Figure 1f(i) the top‐view optical image illustrates this electrode configuration, onto which the NC‐TENG metasurface will be ultimately positioned for subsequent performance measurements and data acquisition. Cross‐sectional image (average thickness of Au layer ≈35 nm) of the metal‐elastomer‐metal structure (Figure 1f(ii)), together with resistance and morphology changes under strain, further confirm the robustness of this nanostructure (Figures S7 and S8 of Supporting Information). The electrode's volume resistance and conductivity are measured to be 0.00012 Ω/cm and 8.3 × 10^5^S/m (for the electrode distance of 0.5 cm, area of 0.0000001 cm^2^). The details of the measurements are given in Table S1 of the Supporting Information.

### 2.2 Optical Characterization of the TENG Metasurface

**FIGURE 2:**
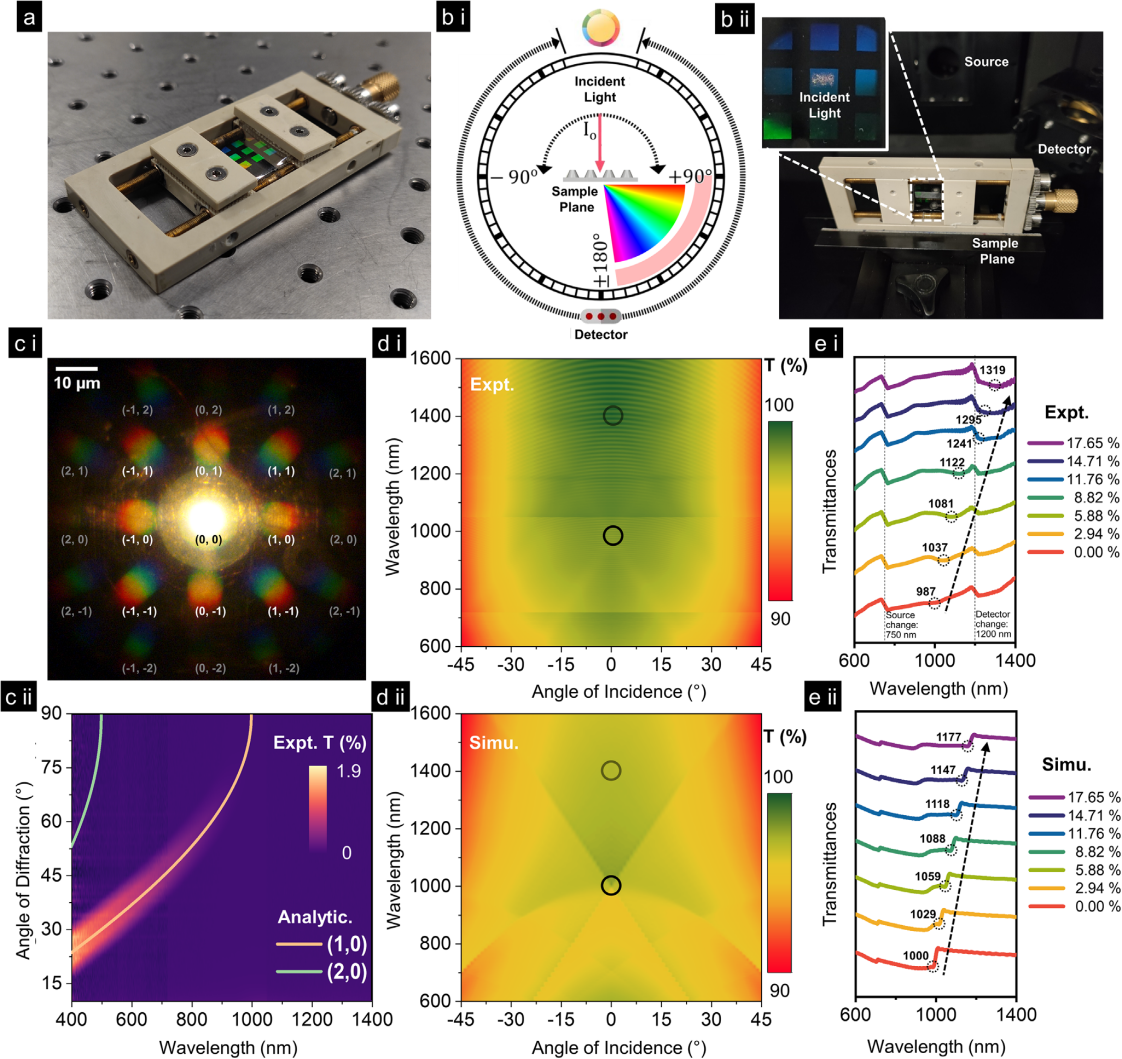
(a) Photograph showing the 100 µm TENG metasurface, attached to a stretching device. Diffraction colors highlight the patterned regions. (b, i) The spectrometer set up for observing the diffraction‐related studies. The detector is moved from 90 to 180 degrees to collect the diffraction orders in transmission. (b, ii) The sample, along with the stretching device, is adjusted to the beam path of the spectrometer. The inset shows the incident source on one of the spatially separated patterned regions. (c, i) 2D diffraction pattern of the TENG metasurface collected under the Bertrand lens setup. Each of the diffraction spots contains multiple wavelengths because of the white light source. (c, ii) for the order (1, 0), wavelength dependency with the diffraction angle is collected experimentally and compared with analytical calculations. (d, i‐ii) Experimental vs simulation comparison of angle/resolved transmittance. The position of the RAs for cover and substrate is highlighted by circles that split on increasing the angle of incidence increases. (e, i‐ii) Using the stretching device, the TENG metasurface is stretched systematically. The position of the RA is red/shifted as seen from both experimental and simulated transmittances.

Figure 2a displays the photographic image of the NC‐TENG metasurface mounted on a stretching device designed to apply mechanical strain. The metasurface, approximately 100 µm thick, exhibits vivid diffraction colors that distinctly delineate the patterned regions, visually confirming the periodic nano‐structuring and its pronounced interaction with incident light. The optical setup employed to investigate these diffraction characteristics is illustrated in Figure 2b, where the spectrometer is precisely aligned to record the diffraction orders generated by the metasurface. In this configuration, the detector arm can be rotated from 90° to 180° around the sample to capture multiple diffraction orders in transmission mode (Figure 2b(i)). The corresponding photograph in Figure 2b(ii) depicts the experimental arrangement, showing the sample mounted on a stretching stage along the optical beam path within the spectrometer system. The inset image highlights light incident directly on a spatially isolated patterned block, underscoring the importance of precise optical alignment to isolate specific structural regions and achieve a high signal‐to‐noise ratio. Moreover, owing to the large patterned area of the metasurface, standard transmittance and absorbance measurements can be employed, eliminating the need for microscale focused‐beam spectroscopy. Figure 2c(i) presents the 2D diffraction pattern obtained using a Bertrand lens configuration, with the corresponding diffraction orders clearly marked. The observation of higher‐order diffraction peaks confirms that the PDMS‐based metasurface possesses a sufficiently high aspect ratio of the nanocones, yielding enhanced diffraction efficiency. This observation indicates that the fabrication process produced not merely surface‐relief gratings, but rather a well‐defined metasurface with distinct optical characteristics. Each diffraction spot exhibits multiple spectral components, arising from the broadband white‐light illumination employed in the measurements. Figure 2c(ii) further illustrates the analysis of the (1,0) diffraction order, where the wavelength dependence on the diffraction angle is experimentally recorded. The measured results show excellent agreement with analytical calculations, validating both the design precision and the optical performance of the fabricated metasurface.

Figure 2d(i,ii) presents a direct comparison between the experimentally measured and simulated angle‐resolved transmittance spectra, revealing distinct Rayleigh anomalies (RAs) corresponding to both the cover medium and the substrate interface. Notably, RA refers to the abrupt spectral feature that appears in the transmission or reflection spectra of a periodically structured surface when one of the diffracted orders transitions from propagating to evanescent. This condition occurs when the in‐plane component of the diffracted wavevector equals that of the incident light; the spectral position of the RA is given by $R{A_{c/s}}^{\pm mth}=\frac{\Lambda }{m}\left(n_{c/s}\mp \sin\theta_{inc}\right)$, where Λ is the periodicity of the grating, *θ*_inc_ is the incident angle, 𝑚 = 1,2, corresponds to the diffraction orders, and n_c_ and n_s_ represent the cover and substrate (glass) refractive indices, respectively. Accordingly, for a lattice periodicity of 1000 nm, the Rayleigh anomaly (RA) modes corresponding to the cover (n_c_ = 1) and substrate (n_s_ = 1.4) are observed at 1000 and 1400 nm, respectively (as indicated by the circles in the graph). Under oblique incidence, these modes become non‐degenerate and progressively diverge with increasing incidence angle. Because of the 2D periodic nature, both classical (linear) and conical (parabolic) diffraction properties can be observed [46]. This behavior highlights the angular sensitivity of the NC‐metasurface, a key characteristic for applications requiring tunable optical responses under varying illumination conditions. Finally, the mechanical tunability of the 2D NC‐metasurface is examined under identical optical conditions by systematically stretching the sample using the stretching device. Both the experimental and simulated transmittance spectra (Figure 2e(i,ii)) reveal a progressive red‐shift of the Rayleigh anomaly (cover) with increasing strain. This spectral shift directly evidences the mechanical flexibility of the metasurface and underscores its potential for strain‐responsive optical devices operating in mechanically dynamic environments.

### 2.3 Triboelectric CE Mechanism and Characterization of Untethered TENGs

**FIGURE 3:**
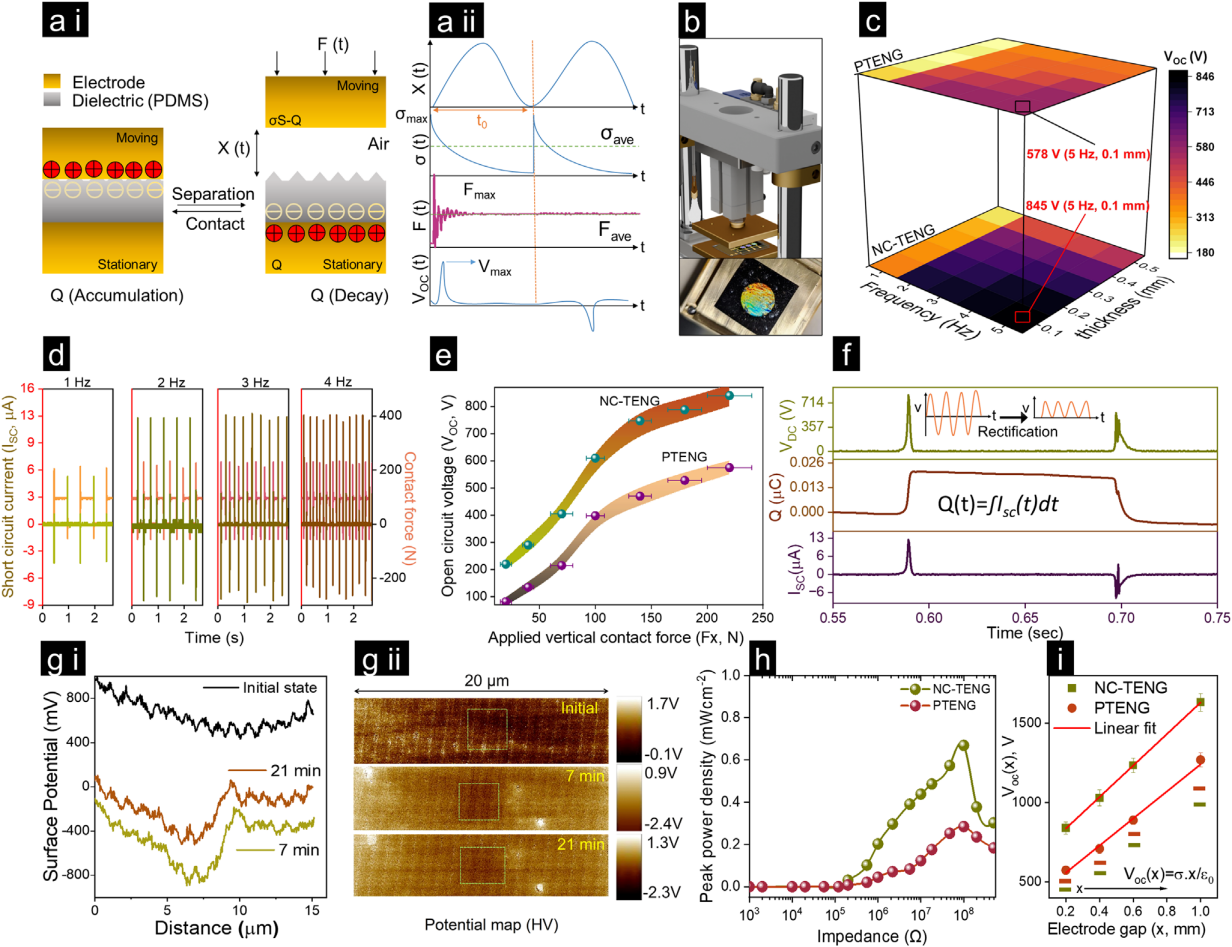
Triboelectric characterization of untethered TENGs (NC‐TENG & PTENG). (a, i) Schematic of the dynamic charge transfer mechanism, illustrating charge accumulation and decay in PDMS‐based TENG metasurface under the contact‐separation mode. The schematic highlights a parallel‐plate capacitor design comprising two electrode (Cu) plates (movable top and static bottom) and one dielectric layer (NC‐TENG) in air. (ii) The contact electrification (CE) process decays exponentially as a function of relaxation time. t_0_ is the transfer time and is related to mechanical motion. The average periodic vertical force (*F*_ave_) is 220 N. *V*_oc_ represents open circuit voltage. (b) Pneumatically‐controlled vertical contact force generator in a twin‐plate electrode configuration. The vertical actuation system is powered by a precision‐controlled pneumatic actuator capable of delivering a maximum vertical contact force of (500 ± 20) N, depending on air pressure. The bottom shows the placement of TENG on the bottom electrode. (c) Color heatmaps for NC‐TENG and PTENG demonstrating the interdependence of the vertical contact‐separation frequency (Hz) and thickness (mm) on the triboelectric output (*V*_oc_, V). (d) Short‐circuit current (*I*_sc_) output of the NC‐TENG at 1–4 Hz illustrates consistent yet frequency‐independent current in the higher frequencies. (e) The *V*_oc_ follows a quasi‐linear increase with dynamic vertical contact force F(x) and tends to saturate at higher thresholds (>100 N). (f) Voltage (*V*_dc_, V), charge (*Q*, µC), and *I*_sc_ (µA) for a single waveform at 5 Hz. (g, i) Kelvin probe atomic force microscopy (KPFM‐AFM) to elucidate surface potential distribution and relaxation process for NC‐TENG, before and after friction, and (ii) corresponding time evolution (0–21 min) potential map. (h) Dependence of output peak power density (mW/cm^2^) on electrical impedance (Ω) for NC‐TENG and PTENG. (i) Linear dependence of *V*_oc_(x) with respect to electrode gap (mm) for both NC‐TENG and PTENG.

In piezoelectric and triboelectric systems, conduction is primarily considered to be capacitive, in which Maxwell's displacement current is the only mechanism of electrical transport, that is, power is transferred not due to the free charge flow between the electrodes, but by electromagnetic wave and induction phenomena [52]. Consequently, output current (i) for TENG would be represented as, $i=\frac{dQ}{dt}=C\frac{dV}{dt}+V\frac{dC}{dt}$, where *Q*, *C*, and *V* represent accumulated charge, capacitance and applied voltage, respectively. The NC‐TENG operates in a contact‐separation mechanism analogous to a parallel‐plate variable capacitor (Figure 3a). In this mode, a dielectric on one electrode comes into and out of contact with an opposing electrode, generating bound triboelectric charges during contact and inducing current upon separation [53]. When the surfaces touch, electrons transfer due to contact electrification, leaving an effective surface charge density σ on the substrate. As the plates separate by a distance *x*, they form a capacitor $C\left(x\right)=\frac{\varepsilon_{0}S}{x+d_{0}}$ (with S the contact area and *d_0_* an effective residual gap equal to the dielectric thickness at full contact). The decreasing capacitance on separation produces a growing electric field and hence an open‐circuit voltage that rises with gap. By Gauss's law, the open‐circuit voltage is given by $V_{OC}\left(x\right)=\frac{\sigma (x+d_{0})}{\varepsilon_{0}}$, which increases approximately linearly with *x*. Under open‐circuit conditions, no charge can flow, so the tribo‐charges remain on the surfaces and *V_OC_* (*x*) reaches a maximum at full separation. In contrast, under short‐circuit conditions, the two electrodes are electrically connected, allowing for induced charge transfer *Q_SC_* (*x*) through the external circuit following separation. The transferred charge at a gap *x* can be obtained from the capacitor relation *Q* = *C*.*V*, so that the electrostatic potential difference is initially zero (contact) and builds as the plates separate. This yields $Q\left(x\right)=\frac{\sigma Sx}{x+d_{0}}$, which starts at zero (when x = 0) and approaches σ*S* as *x* becomes large (full separation). The short‐circuit current is then simply the time derivative of transferred charge, $I_{SC}\left(t\right)=\frac{dQ_{sc}}{dt}=\sigma S\frac{d}{dt}\left(\frac{x}{x+d_{0}}\right)+S\frac{x}{x+d_{0}}\frac{d\sigma }{dt}$. This manifests as the current flow during the moments of increasing or decreasing gap while the system tries to neutralize the time‐varying *V_OC_* by redistributing charge between electrodes [52, 53, 54]. Figure 3a(i) illustrates this dynamic charge transfer model for the NC‐TENG, highlighting the two metal electrode plates (top movable, bottom fixed) and the PDMS dielectric layer arranged in a parallel‐plate capacitor geometry. Figure 3a(ii) further shows how the triboelectric charges generated on the polymer surface during each contact event are not permanently retained at full magnitude. Between successive contact‐separation cycles, the surface charges partially decay with a characteristic relaxation time. However, because the device is operated periodically, each new contact replenishes the lost charge. The time *t*_0_ marked in Figure 3a(ii) denotes the brief interval of charge transfer during contact (essentially the rise time of *Q* when the plates meet), which is dictated by the mechanical friction speed. Figure 3b illustrates the pneumatic system used to apply controlled vertical force to the TENG device. In our experiments, a relatively large average contact force (∼220 N per cycle, with dynamic force exceeding 500 N) is applied to maximize charge generation, ensuring strong contact between the nanocone array and the opposing electrode. Figure 3c presents color heatmap plots of the open‐circuit voltage as a function of the contact‐separation frequency and the PDMS layer thickness, for both NC‐TENG and PTENG (pristine, un‐patterned TENG). At the lowest frequency (1 Hz), the peak *V_OC_* is substantially lower than at a higher frequency (5 Hz). At higher frequencies (3–5 Hz), the interval between contact events is shorter than the charge relaxation time, so the surface retains more charge from cycle to cycle, thereby increasing the average σ and *V_OC_*. However, at significantly higher frequencies, *V_OC_* appeared frequency‐independent, owing to the charge saturation effect. The dielectric thickness also influences the voltage output. In general, a thicker dielectric layer leads to a higher open‐circuit voltage in the parallel‐plate model ($V_{OC}\left(x\right)=\frac{\sigma (x+d_{0})}{\varepsilon_{0}}$). However, increasing the thickness can have competing effects: a thicker dielectric may reduce the capacitance excessively or impede full contact [55]. In our measurements, the thinnest specimen yielded the highest voltages at a given frequency, likely because thinner layers favor closer contact and lower internal impedance without significant dielectric breakdown. At 5 Hz and 0.1 mm thickness, the NC‐TENG achieved an impressive *V_OC_* =  845 V, whereas the PTENG under the same conditions produced 578 V. This corresponds to ∼46% enhancement in *V*_oc_ due to the nanocone architecture. The short‐circuit current behavior with respect to frequency is shown in Figure 3d. Notably, the peak magnitudes of the current pulses remain nearly unchanged as frequency increases (2–4 Hz), which implies that each event transfers approximately the same amount of charge, so the amplitude of each current spike (δ*Q*) over the short transfer time is identical. Beyond 1 Hz, making the cycle faster does not generate more charge per cycle. Thus, the device's per‐cycle charge transfer $Q_{max}∼\sigma$ is relatively constant. To elucidate frequency‐dependent charge and current trend, we extracted time‐resolved current waveforms at 1–4 Hz over a fixed time window (2 s) and computed the RMS Current (*I*_rms,_ to quantify the effective AC output) and average transferred charge per Cycle (*Q*_ave_) using the relation, *Q* = *I*_rms_ × *T*, where, *T* = 1/*f*. Evidently from the Figure S9, *I*_rms_ increases with frequency rising from ∼0.25 µA at 1 Hz to ∼0.92 µA at 4 Hz. This behavior is consistent with the expected scaling of output current in triboelectric generators, where *I*_rms_ is directly proportional to both the transferred charge (*Q*) and the actuation frequency (*I* = *Q* × *f*). The increasing number of contact‐separation cycles per unit time results in a higher net current, even when the charge per cycle is slightly reduced. Average charge per cycle (*Q*_ave_) on the other hand, exhibits a mild decline with increasing frequency. This small but consistent reduction is likely due to reduced contact time and mechanical relaxation at higher frequencies. At elevated actuation speeds, the effective interaction time between triboelectric surfaces is shortened, possibly limiting full charge induction and separation. Moreover, viscoelastic lag in the PDMS layer and incomplete electrical relaxation may also contribute to this trend.

Figure 3e plots the open‐circuit voltage of the NC‐TENG as a function of the applied peak contact force in each iteration. An approximate linear rise of *V_OC_* with increasing force in the low‐to‐moderate force regime is observed, followed by a saturation of forces beyond ∼100 N. This behavior can be understood by the contact mechanics and charge generation. At light forces, only the tips of the nanocone array (or isolated regions of a flat surface) make contact, resulting in a limited true contact area and thus a smaller amount of tribo‐charge deposited. As force increases, the soft PDMS nanocones deform, and more contact area is engaged with the counter surface, thereby generating more charge (higher σ) up to the material's charge saturation limit. Beyond a certain force (∼100 N for our device), the contact area approaches 100% of the available surface, and the PDMS surface charge density itself reaches a maximum (all available charge trap sites are filled, and/or further force cannot create additional charge). To interpret the tactile force mapping efficiently, we have included a calibration chart to convert applied normal force (in N) to average nominal pressure (in kPa), based on the device's macroscopic contact area (Figure S10). While this estimation assumes uniform contact over the nominal, it offers a useful approximation for comparative purposes. The nanocone metasurface is particularly effective in the saturation effect as compared to a PTENG, as it achieves a larger fractional contact area at a given force, which means the NC‐TENG can attain its full‐charge condition at a lower macroscopic force. The theoretical relationships are directly validated by the experimental time‐resolved measurements of voltage, charge, and current for a single cycle of the NC‐TENG, as shown in Figure 3f. Here, the device is driven in a periodic contact‐separation mode at 5 Hz while monitoring the open‐circuit voltage *V_OC_*, transferred charge (*Q*), and short‐circuit current on separate runs. The voltage profile (green curve in Figure 3f) closely follows the plate separation: starting near 0 V at the moment of contact (minimum *x*), then rising steeply as the gap increases, and peaking at around 785 V when the plates reach maximum separation. The *Q* (orange curve) exhibits an inverse behavior: it is near zero at contact (since no net charge has flowed when the plates just touch under open‐circuit conditions), then climbs during separation as electrons are drawn through the external circuit to balance the increasing potential. At maximum gap, *Q* approaches a saturation value corresponding to the total triboelectric charge on the surfaces (here on the order of 0.02 µC, consistent with $Q_{max}∼\sigma S$. When the plates revert to contact, this charge flows back (*Q*∼0). Accordingly, the short‐circuit current is observed as sharp spikes in which a positive magnitude appeared during the separation half‐cycle and a negative upon re‐contact. The *I_SC_* peaks correspond to the timescale of fast charge transfer, in agreement with $I_{SC}\left(t\right)=\frac{dQ_{sc}}{dt}$. The alignment of the *V_OC_*, *Q*(*x*) and *I_SC_* (*t*) demonstrates self‐consistency and is governed by the parallel‐plate capacitor model [52, 53, 54, 55, 56]. To demonstrate the higher overall charge retention rate of NC‐TENG over pristine counterpart (PTENG), we have plotted absolute charge decay *Q*(*t*) and normalized decay curves $\frac{Q(t)}{Q_{0}}$ for both (Figure S11). The plots indicate that the NC‐TENG retains >60% of its charge after 20 s, whereas the PTENG retains only ∼35% in the same duration, quantitatively illustrating the enhanced stability of surface charges on the nanostructured PDMS. The effect of light touch contact force (3 N) on NC‐TENG morphology against pristine PTENG and 1D‐structures further validates that the effective contact area varies linearly with *V*_oc_ (Figure S12). The plot compares real‐time voltage signals of PTENG, 1D‐TENG, and NC‐TENG under continuous tapping at ∼3 N. Output voltage amplitude increases in the order PTENG < 1D‐TENG < NC‐TENG. The NC‐TENG shows stronger and more stable waveforms, confirming that nanocone geometry improves field concentration and effective contact area, enabling more efficient energy conversion from identical mechanical input.

The mechanism and retention of triboelectric charges on the PDMS metasurface are probed by Kelvin probe force microscopy (KPFM), and are shown in Figure 3g. Following the NC‐TENG surface charged by rubbing/contact, KPFM measurements evaluate the surface potential distribution (CPD) on the substrate at various time intervals. Figure 3g(i) shows a representative line profile of the surface potential on the NC‐TENG immediately after contact electrification (t = 0) and after a delay (t ≈ 21 min). Initially, the PDMS surface exhibits distinct potential peaks of several mV; however, it experiences an exponential decay of surface charge. The 2D KPFM maps in Figure 3g(ii) further illustrate this relaxation: right after charging, the nanocone surface has a heterogeneous potential landscape with strong localized charges (hot spots), which gradually relax and fade after 7 and 21 min. Even after 21 min, however, some residual potential remains, implying that not all triboelectric charge has dissipated. These observations reinforce the earlier hypothesis that tribo‐charges have a finite lifetime on the surface (necessitating frequent contact to sustain output) and highlight the charge trapping ability of the nanostructured polymer [17]. The nanocone array, with its high surface area and possibly deeper trap sites (due to surface roughness and polymer interface states), can hold charges for longer periods compared to a smooth surface, which is beneficial for maintaining σ between cycles. In practice, this means the NC‐TENG is less prone to rapid self‐discharging, enabling it to accumulate higher charge over repeated cycles. The impact of the nanocone topology on power output is finally demonstrated in Figure 3h, which plots the electrical peak output power density as a function of load resistance for both. Each device is driven under identical mechanical excitation, and the voltage‐current characteristics are recorded across various resistive impedances (from high resistance for nearly open‐circuit to low resistance for near short‐circuit). Both specimens exhibit a classical TENG behavior. The optimal load for peak power is 100 MΩ, which corresponds to the internal impedance of the TENG. Crucially, the NC‐TENG delivers a substantially higher maximum power than the PTENG at its optimum load. The nanocone‐enhanced device achieves on the order of double the peak power density of the flat device. The PTENG produces 0.32 mW/cm^2^ at its peak, and the NC‐TENG reaches ∼0.7 mW/cm^2^ under the same conditions. This enhancement in power output directly stems from the higher *V_OC_* and sustained charge generation of the NC‐TENG. In fact, considering electrical power, P = V.I, and the NC‐TENG shows ∼1.46× higher *V_OC_* for a similar Q (since the charge per cycle is comparable between NC‐TENG and PTENG, as noted), one would expect the power to improve by roughly 1.46^2^ =  2.13× in the ideal case. The experimental ∼2× increase in peak power is in line with this expectation. However, in TENGs, peak power is not equivalent to the average power delivered over a cycle. A more application‐relevant metric would be the rms (root‐mean‐square) power, which calculates the effective power that a load would experience over many cycles. Therefore, we have carried out rms analyses using the same impedance range and experimental conditions as in Figure 3h. This procedure accounts for the actual pulse shape and cycle of the NC‐TENG and PTENG output and does not rely on any sinusoidal assumption. The resulting RMS power density as a function of load impedance is presented in the supporting information (Figure S13). As expected, NC‐TENG consistently yields higher RMS power density than the PTENG at all impedances. In the output, the NC‐TENG reaches an RMS power density on the order of 2–2.5 µW cm^−^ [2] at its optimum load, whereas the PTENG attains 0.8–1.0 µW cm^−^ [2] at its matching load. As expected for a pulsed, non‐sinusoidal output, the rms power values are lower than the corresponding peak‐power values reported in Figure 3h, but the relative enhancement factor of the NC‐TENG over PTENG is conserved in both cases.

Figure 3i provides a direct confirmation of the parallel‐plate capacitor model by measuring the *V_OC_* as a function of static electrode gap *x* (using mm‐precision separation mechanism) for both NC‐TENG and PTENG. In both cases, *V_OC_* increases linearly with gap distance within the measured range, which is exactly as predicted by $V_{OC}\left(x\right)=\frac{\sigma (x+d_{0})}{\varepsilon_{0}}.$ Extrapolating the linear fit toward *x* = 0 yields a non‐zero intercept, corresponding to $V_{OC}\left(x\right)=\frac{\sigma d_{0}}{\varepsilon_{0}}$. This is the small residual voltage when the surfaces are in full contact, owing to the finite thickness *d_0_* of the dielectric that separates the tribo‐charges. The slope of *V_OC_* (*x*) is proportional to $\frac{\sigma }{\varepsilon_{0}}$, and it is therefore obvious that NC‐TENG's slope is steeper than that of the PTENG. This indicates a higher effective surface charge density σ on the nanocone‐textured surface, quantitatively corroborating that the nanostructures enhance triboelectric charge generation. The linear gap‐dependence validates that, within the operating range, edge effects or field fringing are minimal and the system, in essence, behaves like an ideal parallel‐plate capacitor [54].

Furthermore, it has been observed that ambient water vapor and, in essence, relative humidity (RH) influence charge retention and decay processes. Aging under certain environmental conditions can alter the surface states of TENGs. The electrical outputs are recorded over the span of 15 days under identical experimental conditions and are shown to have no observable variation in *V*_oc_ output (Figure S14a). For humidity variation, we quantified the electrical output under identical RH conditions (22°C, 15%–90% RH) for both NC‐TENG and PTENG (Figure S14b). The *V*_oc_ is recorded for 10 cycles per setpoint, and evidently, it is observed that *V*_oc_ decreases monotonically with RH for both, which is consistent with charge leakage by adsorbed water. The standardized triboelectric outputs are generally recorded at 25% RH, and the humidity effect is found to be reversible after returning to 30% RH. The long‐term cyclic stability tests are also performed and shown in Figure S15. The output *V*_oc_ is observed to be unaffected after 5000 cycles of operations at 3 Hz measurement frequency and apparently negligible reduction in *V*_oc_ (<1.5%) over 5000 cycles of operation, indicating robustness and structural integrity of the metasurface TENGs.

The metal‐elastomer–metal electrode maintains resistance up to ∼80 % uniaxial strain (Figure S7). Under in‐plane stretching of up to ∼15 %, *V_oc_* reduction is <10 % and fully recoverable after relaxation, indicating that the electrode and NC layer accommodate moderate strain without delamination (Figure S16). The strain dependence of the open‐circuit voltage follows a power law dependence according to the relation, *V*_OC_(λ) = *V*_0_λ^−*n*^. The strain‐dependent *V*_oc_ data fitting yields *n*_NC‐TENG_ = 1.31 and *n*_PTENG_ = 0.88, respectively for NC‐TENG and PTENG (Figure S16). The larger exponent for NC‐TENG indicates higher sensitivity to stress‐induced deformation. Uniaxial stretching attenuates the metasurface's field‐enhancing components and reduces effective microcontact area and pressure, whereas the PTENG undergoes predominantly geometry‐induced reduction in *V*_oc_. Consequently, *V*_OC_ decreases faster in NC‐TENG, consistent with the loss of field concentration in addition to the usual capacitance‐increase from dielectric thinning (reduction in effective thickness).

Figure S17 compares the charging characteristics of the NC‐TENG and the pristine PTENG for charging a 10 µF capacitor. The NC‐TENG exhibits a markedly steeper voltage rise with increasing charging cycles, delivering a higher stored voltage at every cycle count. This confirms that the nanocone metasurface enhances effective contact area and local electric field concentration, which enables more efficient charge generation and transfer per cycle. In contrast, PTENG shows a slower and nearly linear charging progression, consistent with lower surface charge density and weaker electrostatic coupling.

### 2.4 Finite Element (FEM) Validation of Metasurface Outputs: Textured vs Pristine

**FIGURE 4:**
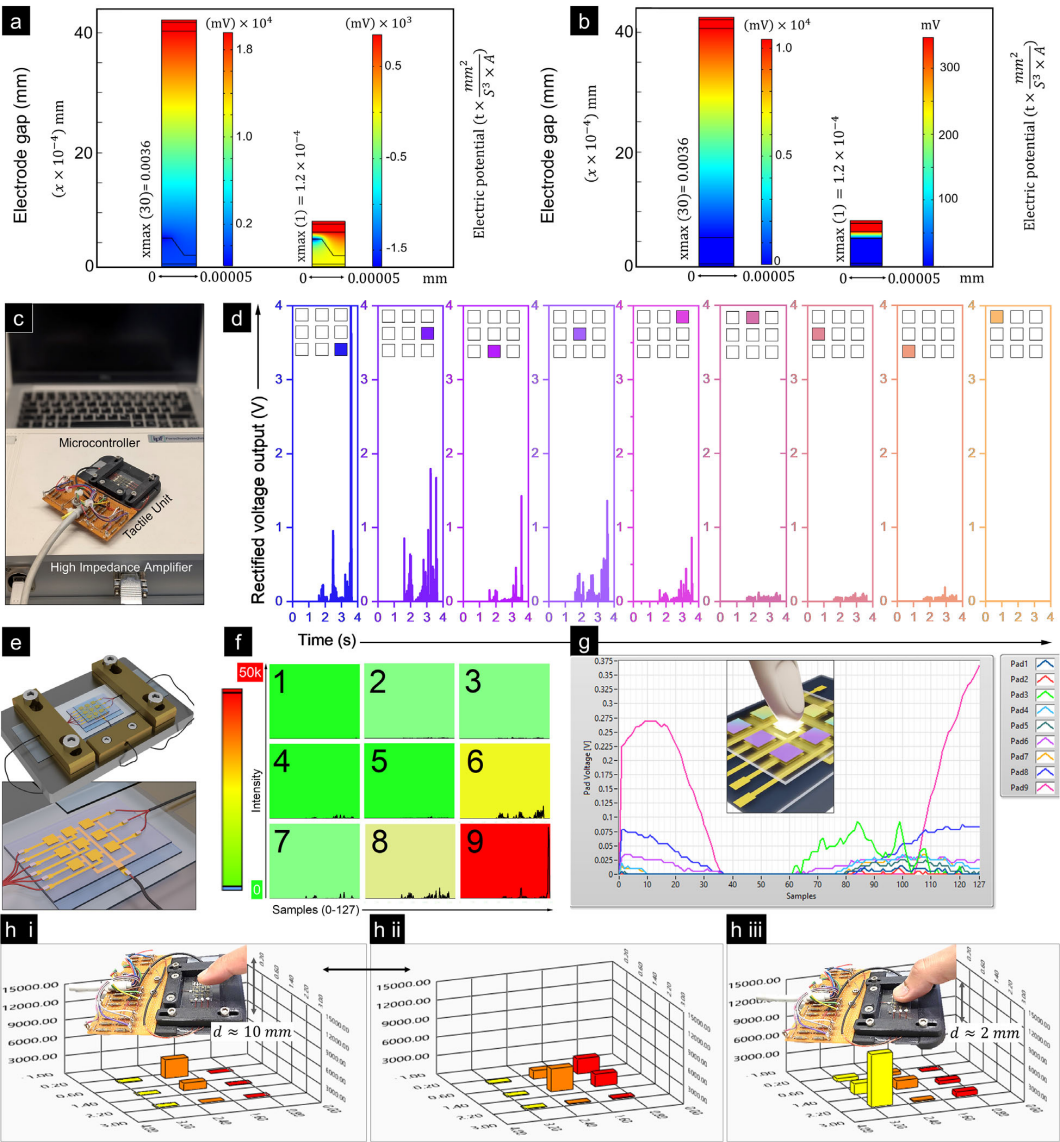
Electrical output characteristics and tactile sensing application of TTAM. Distribution of open circuit potential (mV) of (a) NC‐TENG and (b) PTENG in contact with a flat copper surface at maximum and close‐to‐minimum separation distance of contact surfaces. Potential nonlinear effects for the NC‐TENG in COMSOL‐based finite element modelling can be observed in the distribution of open circuit potential, which is nonlinear and lengthwise non‐uniform, in distinction to linear and lengthwise uniform for the PTENG surface. The finite‐element potential maps are based on the experimentally calibrated surface charge density. (c) Photograph of the triboelectric tactile array matrix (TTAM) architecture, connecting TTAM to the microcontroller and high‐impedance amplifier unit. (d) Real‐time output of a 9‐point tactile matrix when a finger inputs the pad 9, resulting in a voltage signal distribution map on all 9‐points or pads. The corresponding pads are shown in the inset. (e) Schematic illustration of the TTAM unit, attached to a 3D‐printed base for tactile (pressure) demonstration. The top electrode layer highlights the active electrode map that corresponds to each pressure point, while the bottom shows the ground circuit. (f,g) The map of tactile pressure is converted into a digital equivalent of voltage, measured in intensity (a.u.), varying from 1–50k units. A multichannel data acquisition system is utilized for large‐scale data processing and real‐time tactile imaging, employing a synchronous data acquisition card with integrated signal conditioning for multichannel (9‐point) voltage measurements. The image illustrates the tactile pressure mapping in terms of the digital equivalent of output voltage (intensity, arbitrary unit). The voltage shown here is not the actual voltage, but a normalized output, followed by amplification. (h) Demonstration of NC‐TENG TTAM in induction‐based non‐contact position recognition array: (i) the recorded intensity signal is directly correlated to the distance between the triboelectric surface and finger and directly translates into a higher or lower output signal depending on the proximity of the finger to the base plane. (ii) Wider area tracking while hovering the finger over the array points, giving rise to multiple peaks. (iii) The proximity of the finger as close as 2 mm to the base plane translates into a sharper and higher signal intensity.

To assess the potential improvement of the performance of textured vs. flat contact surfaces, the maximum and average power responses are compared under smooth, textured, and homogenized textured surface conditions (Table S2). The responses indicate that an improvement of about 163 % (or 113% for homogenized models) can be predicted for the peak power response of the textured surface, which can be considered as compatible with an increase of about 130 % according to the experimental results (Figure 1h). To this end, it should be noted that certain differences between simulation and experimental results could be due to corresponding differences in gap vs. time functions. Additional insight could be gained by comparing the results for textured vs. homogenized textured PDMS surfaces (Table S2, Figure S18), which indicate that for PDMS layer thicknesses that are significantly larger than the texture size, the increase of open circuit voltage and power density could be less than for nanosized PDMS layer thicknesses. Moreover, since the nominal short circuit charge density of both surfaces differs only by 2 %, the difference in power densities (19 % reduction for homogenized textured vs. textured) can be attributed primarily to differences in the respective open circuit voltages (22 % reduction for homogenized textured vs. textured). Furthermore, it should be noted that for homogenized textured surface, the increase of open circuit voltage vs. smooth surface (46 %) is identical to the increase of respective short circuit charge density, and that the increase multiplier of maximum power density for homogenized textured vs. smooth surface (factor of 2.13) is equal to the square of the respective increase multiplier of short circuit charge density (factor of 1.46), which suggests close to linear voltage and current responses for homogenized and smooth surfaces, and potential nonlinear effects for the textured surface. Further related differences can be observed in the distribution of open circuit potential, which is nonlinear and lengthwise nonuniform for the textured PDMS surface (Figure 4a) in distinction to linear and lengthwise uniform for the flat PDMS surface (Figure 4b). The latter differences may be largely regarded as a result of corresponding differences in the distribution of contact charge.

### 2.5 Triboelectric Tactile Array Matrix (TTAM) and Multiplexed Tactile Imaging

The NC‐TENGs in the 3 × 3 tactile array operate on the coupled effects of contact electrification and electrostatic induction. In essence, each sensing unit behaves like a variable parallel‐plate capacitor whose capacitance depends on the separation *x* between the triboelectric surfaces (the nanocone dielectric) [25]. To first order, *C*(*x*) = ε_0_ε_*r*_ *A*/*x* where *A* is the effective contact area. When a finger touches the sensor, triboelectric charges of equal magnitude and opposite polarity are deposited on the two surfaces. Upon separation of the surfaces (increased gap *x*), a potential difference is generated. The open‐circuit voltage follows: *V_OC_* (*x*) = *Q*/*C*(*x*), where charge, *Q* = σ*A*. As the gap increases, *C*(*x*) drops and ​*V_OC_* (*x*) rises, reaching a maximum. Conversely, pressing the surfaces together (decreasing *x*) increases capacitance and thus lowers the instantaneous *V_OC_* (*x*) [57]. Any change in separation drives charge flow in the external circuit to balance the *V* − *Q* − *x* relation. In short‐circuit condition, this manifests as an induced charge *Q*(*x*) on the electrodes that varies with *x* producing a transient current $I\left(t\right)=\frac{dQ}{dt}$. The polarity of this current depends on the direction of motion (approach vs. separation): when the plates move closer, electrons flow in one direction, and when they move apart, the current reverses direction. The triboelectric tactile array matrix (TTAM) is built by embedding nine NC‐TENG units in a 3 × 3 layout (Figure 4c, e). Each unit consists of a nanocone‐textured triboelectric layer (acting as the friction surface) and Au electrodes. The detailed fabrication process is illustrated in Figure 1e. The NC metasurface on the contact layer dramatically increases the surface area and local field intensity to enhance sensitivity. The nine sensor pixels (each 2 mm × 2 mm) are spaced in a regular matrix with their electrodes routed out to a signal conditioning circuit. A photograph of the assembled 3 × 3 pad and readout electronics is shown in Figure 4c (Figure S19). We employed a high‐impedance buffering circuit for each channel (using FET‐input amplifiers) to accommodate the high source impedance of TENG outputs. These amplifiers (shown in Figure S15, supporting video S1) ensure that TTAM voltage can be measured without significant charge leakage, preserving the integrity of fast transient signals. The conditioned outputs are fed into a multichannel data acquisition (DAQ) system. In our setup, PSoC5‐based DAQ interfaced with a LabVIEW program that handles simultaneous sampling of all nine channels.

The LabVIEW interface is programmed to perform real‐time mapping of the tactile/proximity data from the TTAM. Each channel is software‐mapped to a position in a 3 × 3 grid corresponding to the physical layout of the sensors (labelled 1–9 as in Figure 4f). The system updates the signal from each sensor in real time, effectively multiplexing the data into a live tactile heatmap. In practice, the DAQ rapidly samples each of the nine channels in succession (the sampling rate per channel is high enough to capture the TENG pulses, on the order of kHz, such that the readings are effectively simultaneous for human‐scale events). If the analog signal on one channel exceeds a trigger threshold, the DAQ system captures a defined number of samples on each channel to record the full signal of the complete sensor matrix. The LabVIEW GUI displays the output of each sensor as a colored intensity at the appropriate grid location, and also plots time‐domain waveforms for each channel (Figure 4d). The sharp peaks in Figure 4d correspond to the approach and separation of the finger and, in some cases, minor mechanical rebounds. These signals are reproducible triboelectric pulses rather than electronic noise. The baseline between events returns to zero, confirming the absence of significant drift. This architecture allows the array to detect multiple touch or hover events concurrently since each TENG unit is independently addressed (Figure S20). Simultaneous presses on different pads are recorded as separate signals on those channels without interference. The electrode mapping ensures one‐to‐one correspondence between a sensor and its DAQ channel, which avoids the ghosting issues that can arise in matrix multiplexing of capacitive or resistive touch sensors [58]. The custom interface also implements real‐time amplification and filtering of the TENG signals. We perform digital integration of the signals to obtain a unipolar output proportional to the excitation amplitude (Supporting video S2). This integrated output is involved in producing the color intensity in the mapping (bright colors for strong impacts or close proximity). Therefore, the TTAM device structure and DAQ interface provide a scalable framework for high‐speed tactile data acquisition. Additionally, the triboelectric sensing elements operate in a self‐powered mode, that is, the transduction of mechanical stimuli into electrical signals does not require any external bias or powering of the TENG itself. Therefore, only low‐power readout electronics (DAQ and microcontroller) are used to condition and digitize the generated signals.

Figure 4d shows real‐time voltage signals from all nine channels during a representative sequence of touch events. In this test, pad 9 of the array is tapped. As expected, it generates a distinctive voltage pulse in channel 9 corresponding to the touched pad, along with neighboring pads. Subsequently, pressing pad 9 yields a signal spike on channel 9, followed by simultaneous minor spikes corresponding to neighboring pads. This implies uneven pressure distribution across the tactile array (Figure 4d). The 2D voltage intensity map at a moment when pad 9 is actively pressed is shown in Figure 4f. In that frame, the bottom‐right pad (9) is colored bright orange/red, indicating a high voltage, whereas all other cells stay green/light green (near zero). This demonstrates accurate spatial mapping: the system clearly localizes the touch position and quantifies its relative strength by signal amplitude. We observed a strong correlation between the applied force and the output magnitude. Figure 4g highlights this relationship as it plots the sensor digital output for varying finger forces across the 9 pads. The nanocone surface plays a key role by enabling more contact points under pressure (the cones deform and engage more area), leading to greater charge transfer, in contrast to PTENG‐based TTAM (Figure S20). The response time of the system is on the order of a few tens of milliseconds (limited by the mechanical contact dynamics and the DAQ sampling), which is fast enough for real‐time interaction [59]. The signals in Figure 4d are sharp pulses, each corresponding to the finger tapping on or lifting off a sensor. The output eventually returns to zero between touches, indicating no power draw in the idle state.

### 2.6 TTAM Under Non‐Contact Proximity Sensing

Beyond touch, the TTAM can detect a finger that is hovering above the surface (non‐contact sensing). As the finger approaches the TTAM, it intercepts the electric field from the tribo‐charged surface, effectively increasing the capacitance between the device and ground. This causes the potential difference between the sensor and finger to drop, driving electrons from the device to ground to equilibrate [14, 19]. We demonstrated this by moving a finger at various heights over the array and recording the sensor outputs. Even with no physical contact, the approaching finger altered the electrostatic field of each NC‐TENG, inducing a voltage/current via capacitive coupling. In Figure 4h(i), a finger is positioned about 10 mm above one corner of the array, and only a very small signal is induced. The corresponding 3D output map shows a slight rise (a small bar) at the nearest sensor element, while others remain near zero. As the finger moves closer to the surface (Figure 4h(iii), ∼2 mm gap), the induced voltage on the nearest cell increases dramatically (the bar corresponding to that cell grows much taller, reaching a value over an order of magnitude larger than at 10 mm). This indicates a clear sensitivity to hovering height. The system can thus distinguish a finger that is far against near by the signal amplitude. Additionally, the TTAM demonstrates lateral identification of a hovering object. In Figure 4h, comparing panels (i) and (ii) (which represent the finger at the same height but above two different lateral positions laterally), we observe that the active sensor shifts location according to the finger's position. In Figure 4h(ii), the finger is laterally moved toward the center of the array (indicated by the horizontal arrow), and the output map correspondingly shows a larger signal on a central cell (while the previous corner pad's signal diminishes). Finally, in Figure 4h(iii), the finger is not only closest but also directly over another pad, producing a peak at that pad's location (Figure S21). Through these measurements, we confirm that the array can track a finger's position in the x‐y plane. By analyzing the multi‐channel output pattern at any given time, one can deduce the finger's coordinates. The proximity‐sensing capabilities of the NC‐TENG metasurface e‐skin are further investigated through a series of spatial, temporal, and stability assessments, as detailed in Figure S22 (Supporting Information). A grounded finger positioned 2–10 mm above the metasurface perturbs the near‐field electrostatic environment, generating a distance‐dependent induced voltage *V*_ind_ that monotonically decreases with separation (Figure S22b). Furthermore, pixel‐resolved activation maps reveal that the array can accurately localize single‐point hovering events (Figure S22c), distinguish two simultaneous non‐contact targets (Figure S22d), and resolve complex multi‐point activation patterns across several pads (Figure S22e). These spatial responses arise from the lateral confinement of the tribo‐induced potential fields within the NC‐TENG architecture. Time‐resolved measurements demonstrate rapid approach‐hold‐withdraw detection with response times of 20–40 ms (Figure S22f), enabling real‐time gesture tracking with high temporal fidelity. A 25‐cycle proximity activation sequence (Figure S22g) confirms consistent spatial reconstruction, while a 5‐min stability test using a grounded capacitive stylus (Figure S22h) shows minimal drift (<0.1%) under fixed separation. These outcomes indicate excellent temporal robustness of the proximity mode of TTAM. Therefore, it has been demonstrated that TTAM e‐skin enables reliable non‐contact localization and tracking with high spatial resolution, fast dynamics, and stable long‐duration operation.

### 2.7 Wideband Acoustic Sensing in TTAM‐Based Microphone Patch

**FIGURE 5:**
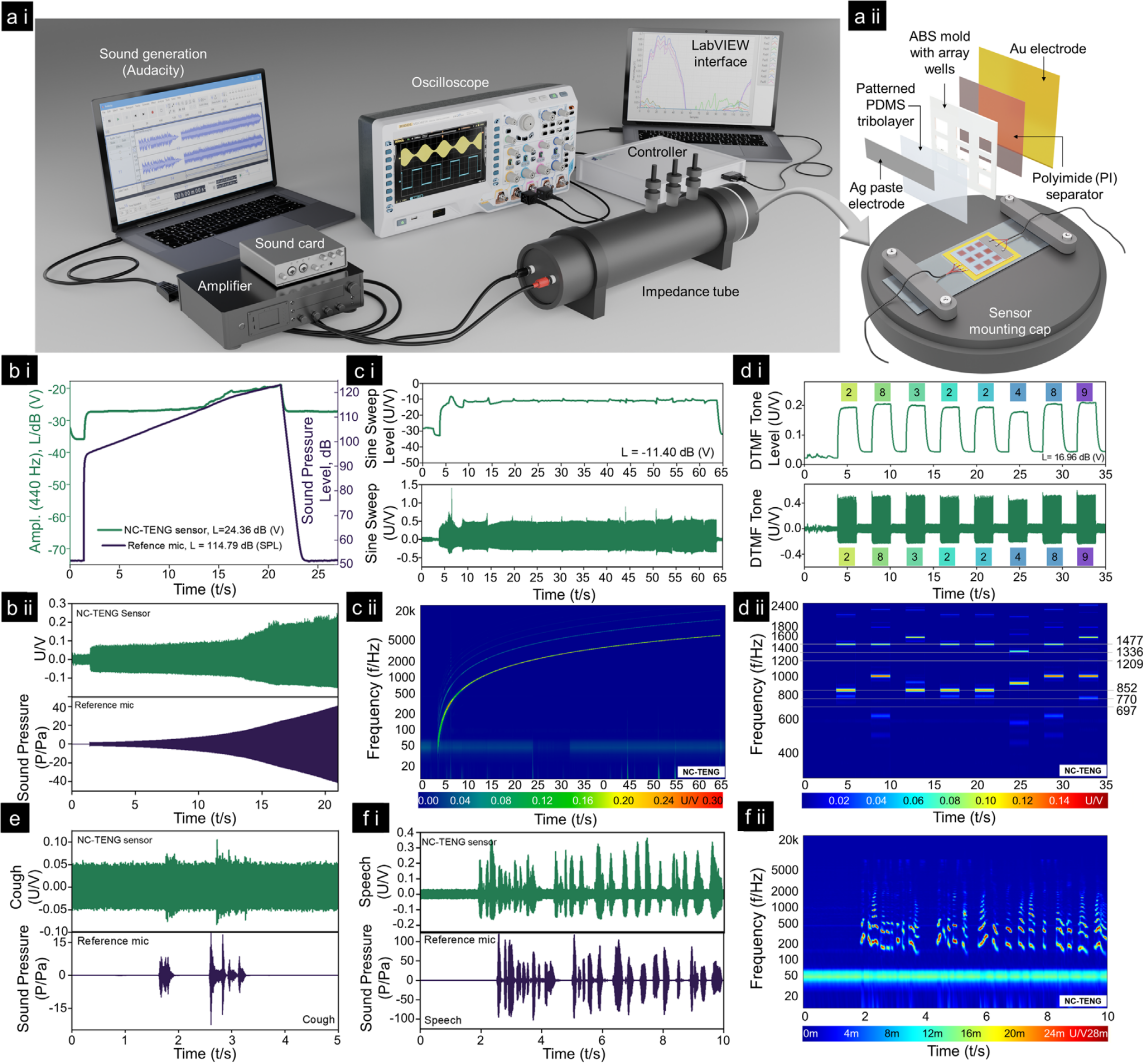
NC‐TENG‐derived self‐powered, thin patch acoustic sensor array. (a, i) Schematic of the experimental setup of the NC‐TENG‐derived acoustic sensor array with all components marked and designated, along with hardware connections. The prototype sensor is placed at the end of an acoustic impedance tube (a, ii) using a 3D‐printed (ABS) mounting apparatus and sealed from the outer regions. The sensor system utilizes a hollow arrayed pattern (3D‐printed), with the active layer placed on top, resembling a diaphragm. Acoustic signals are generated by the laptop with Audacity software and a sound card with a sampling frequency of 44.1 kHz. The generated signals are amplified and sent to the speaker inside the impedance tube. The response of the TENG‐based acoustic sensor is captured, and the measured voltage signals are analyzed. To obtain reference values, a measurement microphone is used in the same position as the sensor. (b, i) Amplitude‐dependent measurements by generating a pure sine wave at 440 Hz with increasing SPL. The measured output for the NC‐TENG sensor (green) and reference mic (violet) is shown. The gentle vs. steep segments reflect different SPL regimes (low vs. high level) rather than instability or non‐monotonic behavior of the sensor. (b, ii) Waveforms of amplitude modulation at 440 Hz by employing a pure sine wave for the NC‐TENG sensor and reference mic. (c, i) Sensor response under a sine sweep (50–6400 Hz). (c, ii) Corresponding spectrogram (FFT). (d, i) Demonstration of the TENG‐based acoustic sensor for the detection of ambient, real‐time sound signals. Detection of the dual‐tone multi‐frequency signaling (DTMF) sound while pressing the numbers (in the order: 2‐8‐3‐2‐2‐4‐8‐9) and the response of the sensor. (d, ii) The corresponding spectrogram illustrates that the NC‐TENG is capable of identifying each dual tone. (e) The NC‐TENG can detect cough noise; corresponding sound waves for NC‐TENG (green) and reference mic (violet). (f, i) Speech pattern recognition (spoken sentence: IPF‐Dresden) using NC‐TENG showed distinct sound waves that are perfectly detectable and matched with reference microphone waveforms. (f, ii) Corresponding FFT spectrogram of the NC‐TENG for speech pattern recognition.

The operating principle of electroacoustic transducers (e.g., microphones) is essentially the inverse of that of loudspeakers, as microphones transform acoustic vibrations into electrical signals [60]. TENG‐based acoustic transducers offer distinct advantages arising from their high surface charge density and, consequently, efficient electromechanical coupling [29]. Unlike conventional microphones, TENG devices are thin‐film, flexible, and skin‐conformable, enabling patch‐type form factors suitable for continuous, wearable applications [61]. As demand for wearable sensing has accelerated in the post‐pandemic world, TENG platforms have become attractive for multimodal detection, including tactile, force, and acoustic stimuli within a single self‐powered architecture. In this context, the 2D metasurface nanocone layer is used as the acoustic interface. The periodic architecture enhances pressure‐charge transduction by increasing effective contact area, concentrating local electric fields, and improving acoustic impedance matching to soft substrates. These effects broaden the usable bandwidth and elevate signal fidelity without sacrificing mechanical compliance. The experimental design of the acoustic sensing experiments is shown in Figure 5a. The sensor prototype is placed at the end of an acoustic impedance tube (Brüel & Kjær‐4206) using a 3D printed mounting apparatus and sealed from the outer regions using clay. This method is selected to generate large‐amplitude directed plane waves [62]. Acoustic signals are generated by using a laptop with Audacity software and a sound card (RME‐ Hammerfall DSP Multiface II) with a sampling frequency of 44 100 Hz. Then generated signals are amplified using an amplifier (Thomann the t.amp) and sent to the speaker inside the impedance tube (Figure S23a,b). In order to have reference values, a measurement microphone (B&K Type 4188 with 31.6 mV/Pa sensitivity) is used in the same position as the sensor. The response of the microphone is captured with Squadriga II (Head Acoustics) front‐end, and the measured signals are analyzed accordingly. Consistent with this design rationale, Figure 5a(ii) demonstrates the composition of the broadband acoustic sensor. Prior to the mechanistic details, the understanding of the design of NC‐TENG is important. The single electrode TENG‐based acoustic patches are designed by stacking multiple layers using a diaphragm design, followed by inserting a top layer triboelectric film with a periodic micro‐pattern (acting as an acoustic metasurface). The hollowed array well mesh below the triboelectric layer (ABS) acts as a dielectric cavity to facilitate acoustic vibration. We effectively create an array of miniature resonators and impedance‐matching elements on the sensor. This design broadens the frequency response and enhances the conversion of pressure fluctuations to electrical charge [21]. In essence, the metasurface provides multi‐resonant pathways that enable the sensor to respond to both low throbbing frequencies and higher frequencies up to a few kilohertz. Such broadband behavior is difficult to attain with a single‐mode membrane; earlier TENG microphones often had to trade off sensitivity at low frequency to gain high‐frequency response or vice versa. In this work, the carefully designed metasurface overcomes that trade‐off, offering high output across a wide spectrum in one device. Moreover, the metasurface STENG (single‐electrode derivative) contributes to signal clarity by focusing and distributing the incoming acoustic energy evenly into the triboelectric active layer, improving the signal‐to‐noise characteristics. The result is a multi‐functional acoustic sensor that is thin (overall thickness of <1 mm), lightweight (0.1 g), wearable, with wideband signal detection (person's cough, voice, environmental audio cues).

We first performed the amplitude dependency measurements on the NC‐TENG sensor. Therefore, a continuous sinusoidal wave at 440 Hz is generated with increasing sound pressure level (SPL). The *V*_oc_ output (measured by the oscilloscope) is directly proportional to the SPL, which is defined as, $SPL\;\left(in\;dB\right)=20log\frac{P}{P_{0}}$, where P and P_0_ represent sound pressure (in Pa), and standard reference sound pressure (2 × 10^−5^ *Pa*), respectively [63, 64]. Measured values from the sensor (green) and the reference microphone (magenta) are compared in Figure 5b(i,ii). It has been observed that the sensor is capable of creating a distinguished output voltage around 112 dB (SPL). The sensor's response is linear over the tested SPL range (∼60–112 dB).

Both NC‐TENG and the reference microphone produce sinusoidal output waveforms whose amplitudes increase linearly with the sound pressure. The NC‐TENG does not show any saturation or compression up to ∼112 dB SPL. It has to be noted that lower SPL levels cannot be accurately identified with NC‐TENG for this frequency. From the calibrated *V*_oc_‐SPL characteristics in Figure 5b, the sensor exhibits a mid‐band electro‐acoustic sensitivity of ≈ 5.3 mV Pa^−^^1^ (≈ −45.6 dB re 1 V Pa^−^^1^), obtained by converting the measured −24.36 dB(V) level into linear voltage and dividing by the reference‐microphone‐derived pressure (10.9–13.9 Pa at 440 Hz). Following this test, a sine sweep between 50 Hz and 6400 Hz is applied. The response of the sensor is given in Figure 5c, and it can be seen that the sensor gave a continuous voltage signal for the whole frequency range. This enables reliable detection of both low‐frequency vibrations and mid‐band audio signals. This performance can be favorably compared to recent TENG‐based acoustic sensors reported in the literature (Table S3 of the supporting information). The wideband ≈50–6400 Hz operability is broader than several earlier triboelectric microphones that often focused on narrower bands. For instance, triboelectric stethoscopes optimized for cardiac auscultation emphasize extreme sensitivity in the low‐frequency range (<500 Hz) but do not cover higher frequencies. Hui et al. achieved an ultrahigh ∼1215 mV Pa^−1^ sensitivity, and ∼36 dB signal‐to‐noise ratio (SNR) for heart sounds using a triboelectric stethoscope, vastly outperforming a piezoelectric stethoscope in detecting faint heart murmurs [60]. However, that device's acoustic bandwidth is inherently limited to the sub‐kilohertz regime of heart acoustics. In contrast, the present metasurface design provides a multi‐octave response covering speech frequencies and beyond. It approaches the performance of broadband TENG auditory sensors that utilize nanofiber membranes or resonator arrays to achieve 20 Hz–20 kHz coverage [31, 32]. Our device's sensitivity is lower than that of the specialized triboelectric stethoscope by Hui et al. NC‐TENG achieves a broad frequency response (∼50 Hz to 6.4 kHz) at the expense of some sensitivity, yet it is still sufficiently sensitive for practical purposes (detecting normal speech, music, ambient noises without any external amplification). The measured sensitivity (5–5.5 mV/Pa at 440 Hz) corresponds to about −45.6 dB (ref 1 V/Pa), which is comparable with recently reported broadband TENG microphones. For example, Sun et al. (2024) reported a nanofibrous TENG microphone covering the full human hearing range with calibrated sensitivity (∼ −50 dB ref 1 V/Pa) sufficient for voice control applications [31]. While our device does not reach ultrasonic frequencies, the tested ∼6.4 kHz upper limit already exceeds the voice‐band range addressed by many triboelectric voice sensors (often <1–3 kHz). Additionally, the NC‐TENG delivers a strong output *V*_oc_ (± ≈0.5 *V*) under acoustic stimuli. Recent design improvements have also contributed significantly to TENG microphone output via structural innovations. Our metasurface approach similarly exploits structural enhancements (periodic nanocone patterns) to boost acoustic‐mechanical coupling, yielding output levels and noise performance on par with these state‐of‐the‐art sensors.

To validate the NC‐TENG's credibility as a real‐time microphone, a set of real‐life sound tests is conducted. First, Dual‐tone multi‐frequency signaling (DTMF) sounds are generated (Figure 5d). Those are the tones that are heard on the telephones when numbers are pressed. The tones that correspond to 2 8 3 2 2 4 8 9 (in that order) are created in the selected case, and the response of the sensor is captured. Under pure‐tone excitation across this range, the NC‐TENG produces distinct voltage outputs at each tone (corresponding to each constituent frequency couple), indicating a consistent sensitivity. Moreover, output waveforms closely resemble the input acoustic waves, which is attributable to high signal fidelity and low distortion over the entire operating band (Figure 5d(ii)). Similarly, transient audio events such as a human cough or spoken words are rendered with sufficient resolution for waveform analyses. The sensor's response to a cough is compared with the reference microphone signal, and the results are shown in Figure 5e. The NC‐TENG device registers the short, bursty waveform of a cough with clear peaks, suggesting possible utility in respiratory monitoring or smart wearable health patches. Compared to conventional microphones, the triboelectric sensor's passive, power‐free nature and high sensitivity to gentle mechanical vibrations offer an advantage in detecting such biomechanical sounds. Additionally, a voice recording of a speech is used. The results are presented in Figure 5f. The spectral content of spoken words (covering a range between 100 Hz and 3000 Hz) is well‐resolved by our sensor, enabling potential voice‐recognition or throat‐mounted microphone applications (Figure S23c). This capability aligns with recent human‐machine interface demonstrations using TENG vocal sensors for speech recognition [65]. The speech pattern in Figure 5f(i) demonstrates a speech signal‐to‐noise ratio (SNR) of ≈33 dB (≈24 dB averaged over the full 10 s segment), confirming that the device provides sufficient acoustic contrast for speech and ambient sound detection without external amplification. Therefore, it is observed that the sensor's response is sufficient to distinguish the components of the respective acoustic scenes. These results demonstrate the device's ability to handle complex acoustic inputs and could be expanded for wireless communication interfaces. There is non‐fluctuating noise in the measurements around 50 Hz, but these types of defects can be eliminated in engineering applications with appropriate circuit designs and signal processing algorithms by using a high‐pass filter.

This work suggests that the metasurface TENG can serve as a wearable acoustic transducer for diverse signals ranging from human physiological sounds to digital audio codes. Based on the NC‐TENG sensor's performance even without external power or elaborate circuitry, it can be concluded that the metasurface‐based NC‐TENG extends triboelectric sensing into true wideband acoustics matching and, in some aspects, exceeding the performance of recently reported TENG acoustic sensors [31, 32, 65]. Table S3 summarizes the key parameters (frequency range, sensitivity, SPL range) of recently published reports highlighting state‐of‐the‐art TAS and compares with those of the NC‐TENG acoustic patch. It combines the broad response range and high signal fidelity needed for complex audio signals with the inherent advantages of triboelectric devices (flexibility, self‐powering, and simplicity). These attributes make it promising for wearable acoustic applications such as voice‐activated interfaces, health monitoring (e.g., breathing or cough analysis), and environmental sound detection. However, there remain practical considerations to address the current bandwidth of 50–6400 Hz, which, while impressive, does not capture the extreme ends of the audible spectrum achieved by some nanoengineered TENG sensors. Nevertheless, by utilizing a dielectric metasurface architecture, the device achieves a notable balance of wide frequency coverage, sensitivity, and versatility that represents a significant step forward for triboelectric acoustic sensing in real‐world scenarios.

## 3 Conclusion

In summary, we have developed a large‐area dielectric metasurface‐based TENG e‐skin that uniquely integrates tactile, proximity, and acoustic sensing into a single flexible platform. The materials design involves a 2D nanocone metasurface in a thin PDMS film, which enhances electromechanical coupling by increasing the contact area and surface charge density at the triboelectric interface. This nanostructured dielectric layer, fabricated via scalable laser interference lithography and soft molding, boosts the electrical output (∼46% higher voltage than a flat surface under identical pressure) and also imparts an optical diffraction signature useful for device characterization. The metasurface TENG is incorporated into a pixelated 3 × 3 array with a stretchable metal‐elastomer‐metal electrode architecture, forming an ultrathin (<0.4 mm) and conformable sensor patch. Benefiting from this unique architecture, the system demonstrates trimodal sensing capabilities beyond those of conventional e‐skins. In tactile mode, the TTAM can map pressure distributions in real time with low inter‐pixel interference, enabling high‐resolution touch imaging. In proximity mode, it detects approaching charged objects (such as fingertips) without direct contact, effectively recognizing gestures and motion in a touchless manner. In acoustic mode, the same device functions as a broadband self‐powered microphone, capturing sound vibrations across a wide frequency range (spanning voice and ambient sound frequencies). The metasurface design contributes to a broad acoustic bandwidth and improved signal fidelity by coupling mechanical vibrations into electrical charge efficiently across low and high frequencies. Together, the tri‐modal e‐skin can sense pressure, gestures, and sound concurrently, with differentiation accomplished via signal processing, highlighting its versatility for future multimodal interfaces.

## 4 Experimental Section

### 4.1 Laser Interference Lithography for Creating Patterned Masters

To fabricate patterned masters via laser interference lithography, microscopic glass slides (25 mm × 75 mm) were first sectioned and cleansed using a solution of isopropyl alcohol, ethanol, and Millipore DI water. These slides were subsequently coated with ma‐N 405 (MicroChem) negative photoresist at 3000 rpm and an acceleration of 1000 rpm/s for 33 s to achieve a uniform layer approximately 500 nm in thickness, followed by prebaking at 105°C to enhance the photoresist's adhesion and stability. Patterning was performed using a helium‐cadmium (He‐Cd) continuous‐wave laser (IK5351R‐D, Kimmon Koha) operating at 325 nm. The laser beam, after passing through a spatial filtering setup comprising a microscope objective, a pinhole, and a collimating lens, was split and directed by mirrors to create interference on the photoresist‐coated substrates. Critical to this setup were the parameters of mirror distance (d) and the distance from the midpoint to the sample stage (z), which determined the interference angle, θ = tan−1(d/2z), and consequently the pattern periodicity, Λ = λ/2 sin(θ), by adjusting “z”. Double exposures of 5 s each at 220 µW were employed, followed by a 60‐s development process to create the patterned photoresist (PR) layer. This PR layer served as the master for subsequent PDMS thin film fabrication. An electronic shutter controlled the exposure dosage, and the substrates were positioned on a rotational stage for azimuthal rotation by 90 degrees between exposures to establish the 2D pattern. Additionally, a 3D printed mask having an array of squares in 3 × 3 geometry (each side, 2 mm; distance between two squares, 1 mm) was used to generate spatially separated arrays, ensuring precise control over pattern dimensions and periodicity for successful master fabrication.

### 4.2 Pattern Transfer on PDMS Thin‐Films

After the fabrication of photoresist masters, the inverse structures were replicated using an elastomeric silicone kit (Sylgard 184, Dow Chemicals, USA) at a prepolymer to catalyst ratio of 10:1 to form the PDMS metasurface. The mechanical properties of the silicone (PDMS) employed here are shown in Table S4. To achieve a minimum PDMS film thickness of approximately 100 microns, the patterned surface was oriented face down on a plastic Petri dish prior to the addition of the uncured PDMS elastomer. The desiccation process was employed to expel air bubbles from the mixture, which also facilitated the removal of trapped air beneath the masters, thereby ensuring complete contact between the patterned surface and the Petri dish. An extended desiccation duration of 1 h was critical to eliminate the air gap beneath the substrates and to allow the viscous PDMS to infiltrate the fine gaps. Following desiccation, the samples in the Petri dish were subjected to a thermal curing process at 180°C for 3 h. The careful detachment of the resist masters from the Petri dish confirmed the formation of an additional PDMS layer on the patterned surface. Using tweezers, the PDMS thin films with replicated patterns were delicately peeled off and stored for subsequent characterization.

### 4.3 Optical Microscopy

A bright‐field reflection microscopy setup utilizing a Nikon ECLIPSE LV100ND microscope was employed to acquire surface images of the PDMS nanocone‐based metasurface. Illumination was provided by a 50 W, 12 V halogen light source focused through a 50× objective lens for unpolarized light exposure. Images were captured using a Nikon DS‐Fi2 5.24 megapixel charged‐couple device camera connected to a computer. Additionally, a Bertrand lens setup in conjunction with a 50× objective lens was utilized to record the diffraction patterns from the grating samples.

### 4.4 UV–vis‐NIR Spectroscopy

UV–vis‐NIR spectroscopy was executed in the transmission geometry depending on using a Cary 5000 spectrometer (Agilent Technologies, USA) coupled with the universal measurement accessory (UMA) to capture the angle‐dependent optical spectra, reported in this article. The beam spot size was fixed to ≈ 5 × 5 mm^2^ for UV–vis and NIR detectors. The dispersion spectra were captured by fixing the detector at 180 degrees and rotating the sample plane from −45 degrees to 45 degrees, whereas the angle dependency of the diffracted orders was carried out by keeping the sample plane fixed at 0 degrees and scanning the detector from 90 degrees to 180 degrees. The measured data were corrected from the spectra of the blank PDMS.

### 4.5 Finite‐Difference Time‐Domain Simulations

For the FDTD calculation of photonic diffraction modes, a commercial‐grade electromagnetic solver from Ansys Lumerical [66] has been utilized with a broadband plane wave source (400 to 1000 nm) with frequency points set equal to that of the wavelength range. Monitor boxes (transmission monitors normally maintained on the substrate) were used to obtain the optical responses of the systems. To implement the metasurface array, periodic boundary conditions were applied in the X and Y directions with perfectly matching layers along the Z axis. An auto non‐uniform mesh with a minimum mesh step of 0.25 nm is selected for all the simulations, with an additional mesh overlay of 2 nm around the nanocones. To identify the diffraction orders, angular‐resolved diffraction modes were obtained using BFAST sources for variation of incidence angles from −45 to 45 degrees, in steps of 1 degree. All simulations reached an automatic stop of at least 10^−7^ before reaching a simulation time of 300 fs.

### 4.6 Finite Element Calculations of the TENG Outputs and Charge Distribution

The theoretical, resistance‐optimized power responses were estimated using the voltage‐charge‐displacement relation according to references [55, 67, 68], adapted to the current structures by defining the *V_OC_* (*x*) and *Q_SC_* (*x*) relations (where *V_OC_* is open circuit voltage, *Q_SC_* is short circuit charge, *x* refers to contact‐separation distance coordinate) based on the results of electrostatic Finite Element Method simulations using COMSOL [69]. A surface charge density σ_0_ on the PDMS is calculated from the experimentally measured *V*_oc_ at small separation using the parallel‐plate approximation (Equation 1), and this *σ*_0_ is applied uniformly on the PDMS surface. Specifically, an axisymmetric structure was employed to represent a single pillar of a textured PDMS surface against a flat copper surface, subsequently converting the obtained performance estimates to those of a periodic unit of the textured surface. Thereby, open circuit potential distributions and the required scalar quantities were obtained for a nanotextured PDMS surface with contact‐charged flat top part of the surface against a metallic contact surface with an oppositely charged segment, assuming that material or ion‐based charge transfer has taken place. Further, a 2D, periodic structure was used for obtaining the estimates for a flat PDMS contact surface. For dimensional compatibility, the total thickness of the PDMS layer of both structures was set equal to 150 % of the height of the nanotexture (450 nm). For an assessment of size effects, additional simulations were performed with flat contact surfaces representing homogenized textured surfaces and equivalent surface charge densities of textured surfaces. For defining the simulated materials, the dielectric constant of PDMS value of 2.4, was applied [70], whereas a value of 10^6^ was assumed as an approximation of the infinity value of dielectric constant for metals. In addition, the constitutive relation of surface charge density (*σ*_0_) vs. contact pressure was obtained from the experimental values of open circuit voltage at minimum gap distance (Figure 1i) using a 1D approximation [55] equation (1). It should be noted, however, that the experimental *V_OC_* at *x* = 0 not being equal to 0, as equation (1) suggests, could be an indication of nonlinearity of the relation at lower *x* values.

(1) $$\sigma_{0}=V_{oc}\left(x\right).\varepsilon_{0}/x$$

where *ε*_0_ ‐ vacuum permittivity.

For assessing the dynamic responses of the surfaces, a smoothed step separation displacement function *x*(*t*) was defined for a duration of 0.025 ms (sample response shown in Figure S18):

(2) $$x=x_{\max}\left(\frac{1}{2}-\frac{1}{2}cos\left(\frac{\pi v}{x_{\max}}t\right)\right),\;\left(t<\frac{x_{\max}}{v}\right)$$

$$x=x_{\max},\;\left(t\geq \frac{x_{\max}}{v}\right)$$

where *v*—nominal velocity of vertical separation of contact surfaces (0.3 m/s applied). For the simulations, an *x*_max_ value of 3.56 µm was employed as an approximation of separation that would correspond to 95 % of the saturation value of short circuit charge density according to the 1D approximation [55, 67, 68].

### 4.7 Triboelectric Measurements

The triboelectric characterization of the NC‐TENG and PTENG was carried out using an in‐house developed vertical contact‐separation setup driven by a pneumatic actuator (Festo GmbH), capable of delivering a maximum vertical contact force of (500 ± 20) N depending on air pressure. For the measurements shown in Figure 3b–f, the contact force was held constant at (200 ± 20) N, corresponding to 3 bar of compressed air, unless otherwise specified. This ensured consistent contact area and triboelectric interaction between the patterned PDMS and electrode (Cu plate) layer across repeated cycles. In the setup (Figure 3b and Figure S24), the bottom plate was stationary (TENG specimen attached to this plate) while the top plate was connected to a pneumatic arm and was controlled with respect to the magnitude of force, frequency, and contact time. The test setup includes a 3‐channel oscilloscope (Rigol DS4024 Digital Storage Oscilloscope), where Ch1 monitors the trigger pulse (indicating cycle timing), Ch2 records the open‐circuit voltage (*V*_oc_), and Ch3 captures the contact force (in N), measured using an in‐line force sensor. The signals were simultaneously streamed to a custom LabVIEW interface via a National Instruments NI‐9239 data acquisition system coupled to a USB carrier. This configuration allowed synchronized, real‐time acquisition and visualization of the mechanical and electrical response parameters (*V*_oc_, *I*_sc_, *Q*) alongside the corresponding mechanical input, including actuation frequency, displacement, and applied force. For charge storage measurements, TENGs were connected to a full‐wave bridge rectifier for ceramic resistors ranging from 1 kΩ to 100 MΩ.

### 4.8 Acoustic Measurements

Acoustic measurements were conducted using an impedance tube (Brüel & Kjær‐4206) and Audacity sound generator software. Acoustic signals with frequencies ranging from 50 to 6400 Hz were generated. The TENG‐metasurface was positioned at one end of the impedance tube, with its leads connected to the probes of an LCR meter. To ensure high directivity of the acoustic signals, the PDMS metasurface was affixed to a 3D‐printed mold that maintained its position around the circumference. The diameter of this mold was tailored to match the 100 mm diameter of the impedance tube. Initially, the response to a click track signal is recorded, demonstrating a relative resistance change exceeding 0.10%. Additionally, DTMF signals in between frequencies of 697 Hz and 1477 Hz are used to assess sensor performance.

### 4.9 Statistical Analysis

All experiments were performed under controlled laboratory conditions, and each measurement was repeated a minimum of three times unless otherwise stated. Electrical output parameters including open‐circuit voltage (*V*_OC_), short‐circuit current (*I*_SC_), transferred charge (*Q*), peak and RMS power density, and impedance‐dependent characteristics were recorded using a 3‐channel digital oscilloscope coupled to a LabVIEW‐based acquisition interface. Reported values represent mean ± standard deviation (SD). Time‐resolved current signals were numerically integrated using Simpson's rule to extract transferred charge per cycle. RMS current and RMS power were computed directly from experimentally acquired waveforms without sinusoidal assumptions. Data processing was carried out in OriginPro 2023 and MS Excel. For triboelectric characterization maps, color heatmaps were derived from averages of ≥10 cycles per state. Force‐dependent measurements used a pneumatic actuator with calibrated load (N) readings. Pressures (kPa) were calculated from macroscopic contact area. Frequency‐dependent statistics (1–5 Hz) were evaluated from multiple 2‐s sampling windows. KPFM measurements were conducted on multiple spatial regions (n ≥ 5) per specimen to extract mean contact potential difference (CPD), relaxation curves, and potential distributions. Optical diffraction and angle‐resolved transmittance data were obtained by averaging three independent spectral scans per configuration. Rayleigh anomaly positions were extracted from peak fitting of transmittance minima, with statistical variation reported as peak position SD. Acoustic measurements were repeated across three independent impedance‐tube mountings. Frequency response spectra (50–6400 Hz) were averaged over five sequential sweeps. SPL‐to‐voltage transfer functions were calculated relative to a calibrated reference microphone. For the tactile array and proximity mapping experiments, each pad response was sampled over ≥10 repeated touch/hover events. Spatial activation matrices were generated from cycle‐averaged rectified outputs, while response times were extracted from rising‐edge fits across multiple trials. Signal intensity values represent normalized and averaged digital outputs (arbitrary units) following identical amplification conditions. Finite‐element simulations used experimentally measured surface charge densities as input parameters. Model outputs (open‐circuit potential, power density, impedance response) were compared with experimentally measured mean values, deviations were quantified through relative error analyses.

Long‐term stability, cyclic durability, and humidity‐dependent tests were evaluated from ≥500–5000 cycles, with performance drift reported as relative percentage change from baseline values.

## Conflicts of Interest

The authors declare no conflict of interest.

## Supporting information




**Supporting File 1**: adma72507‐sup‐0001‐SuppMat.pdf.

**Supplemental File 2**: adma72507‐sup‐0002‐VideoS1.mp4.

**Supplemental File 3**: adma72507‐sup‐0003‐VideoS2.mp4.

## Data Availability

The data that support the findings of this study are available from the corresponding author upon reasonable request.
