# Supplementary material for: Large‐Area 2D Metasurface‐Based Triboelectric E‐Skin Arrays: Contact & Proximity Tactile Mapping with Broadband Acoustic Readouts
Source: Adv Mater. 2026 Feb 11;38(36):e21525. doi: 10.1002/adma.202521525 (PMC13310109; doi:10.1002/adma.202521525)
Supplement: Supplementary file 1 — Supporting File 1: adma72507‐sup‐0001‐SuppMat.pdf. [file ADMA-38-e21525-s001.pdf]

Supporting Information

Large-Area 2D Metasurface-based Triboelectric E-Skin Arrays: Contact & Proximity Tactile Mapping with Broadband Acoustic Readouts

Injamamul Arief[1,*,‡], *Swagato Sarkar*[2,‡], *Anik Kumar Ghosh*[1], *Osvalds Verners*[3], *Kamal Kumar Meena*[1], *Su-Hyeong Lee*[4], *Soosang Chae*[2,4], *Tobias A. F. König*[2,5,6], *Beate Krause*[7], *Andreas Fery*[2,8], *Mehmet Sait Özer*[9], *Anindya Nag*[9,10], *Amit Das*[1]

[1] Department of Elastomers, Division Polymer Materials Engineering, Leibniz Institute of Polymer Research Dresden, Hohe Straße 6, 01069 Dresden, Germany

[2] Department of Functional Colloidal Materials, Division Physical Chemistry and Physics of Polymers, Leibniz Institute of Polymer Research Dresden, Hohe Straße 6, 01069 Dresden, Germany

[3] Institute of Materials and Surface Engineering, Faculty of Natural Sciences and Technologies, Riga Technical University, Paula Valdena Street 7, LV-1048 Riga, Latvia

[4] School of Energy Materials and Chemical Engineering, Korea University of Technology and Education, 31253 Cheonan, South Korea

[5] Faculty of Chemistry and Food Chemistry, Technische Universität Dresden, Bergstraße 66, 01069 Dresden, Germany

[6] Dresden Center for Intelligent Materials (DCIM), Technische Universität Dresden, Hallwachsstraße 3, 01069 Dresden, Germany

[7] Department of Sustainable Polymer Structures, Division Macromolecular Chemistry, Leibniz Institute of Polymer Research Dresden, Hohe Straße 6, 01069 Dresden, Germany

[8] Chair for Physical Chemistry of Polymeric Materials, Technische Universität Dresden, Mommsenstraße 4, 01062 Dresden, Germany

[9] Faculty of Electrical and Computer Engineering, Technische Universität Dresden, Helmholtzstraße 10, 01069 Dresden, Germany

[10] Centre for Tactile Internet with Human-in-the-Loop (CeTI), Technische Universität Dresden, Nöthnitzer Straße 46, 01187 Dresden, Germany

‡ Equal contribution
* Corresponding author
Email: arief@ipfdd.de

Experimental Setup for Laser Interference Lithography (LIL)

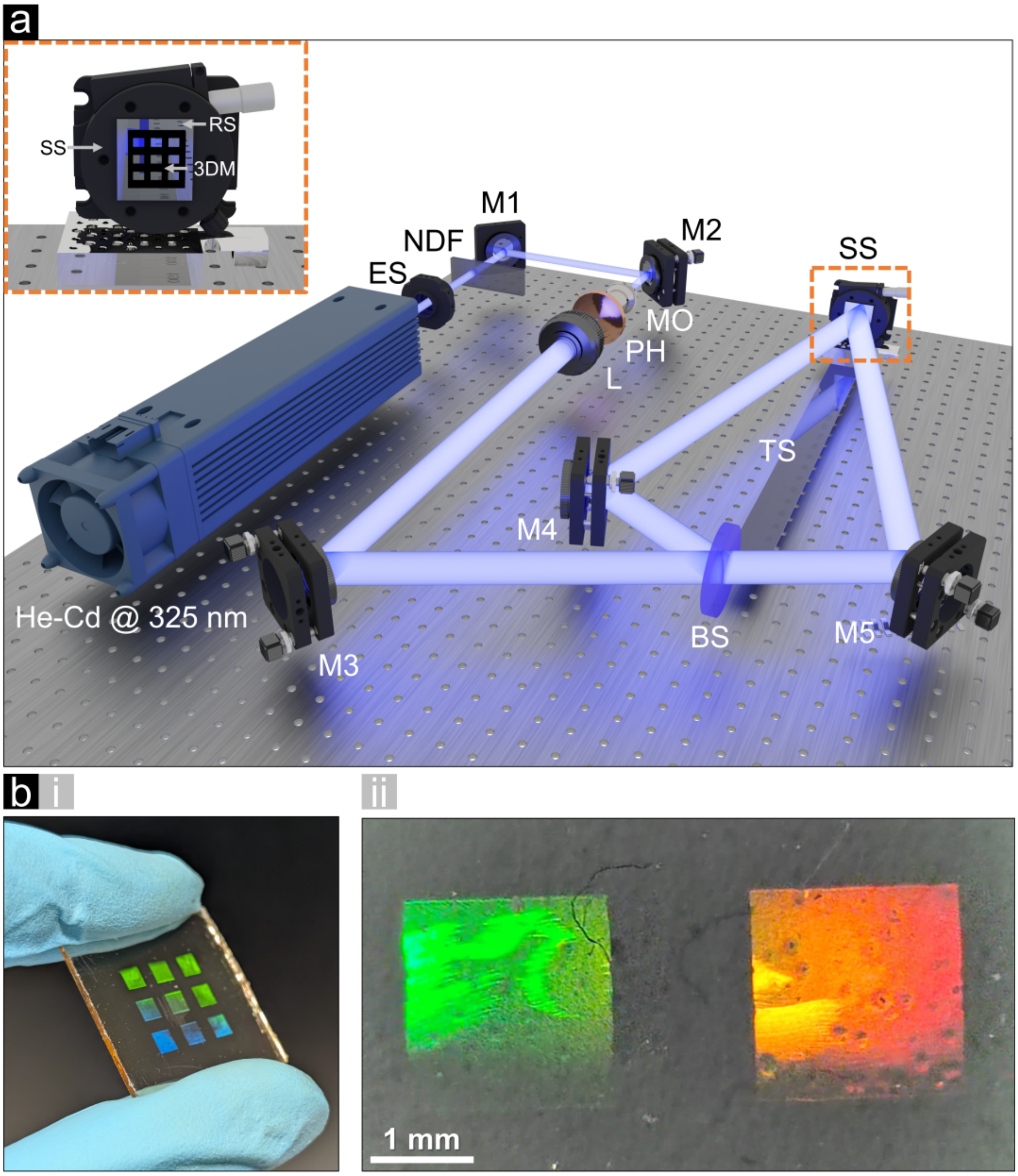

Figure S1. (a) Schematic of the experimental setup for Laser Interference Lithography. He-Cd: Helium Cadmium Laser; NDF: ND Filter; M1, M2, M3, M4, M5: Plane mirrors, MO: Microscope Objective; PH: pin-hole; L: Collimating Lens; BS: Beam splitter; TS: Translation stage, SS: Sample stage. Inset shows the SS with resist-coated susbtrate (RS) and 3D-printed mask (3DM). (b.i) 2D arrays comprising a 3×3 matrix formed after removal of overexposed photoresist regions. (b.ii) Magnified view showing the distinction between patterned and unpatterned regions.

AFM Details of the Photoresist Master Showing Holes

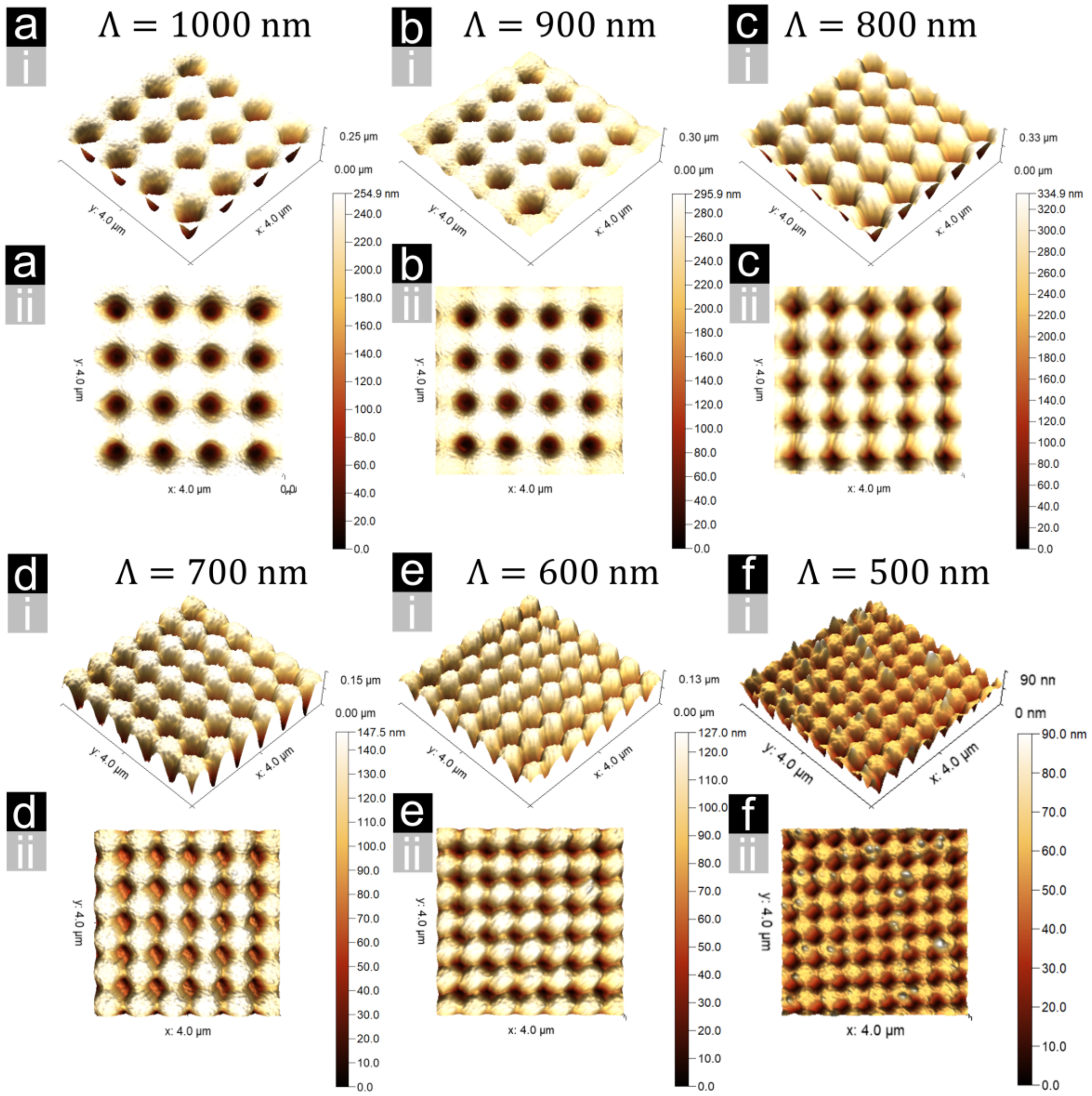

Figure S2. (a-f) AFM profiles of PR patterns on a glass substrate with periodicities from 1000 to 500 nm. Top row, panels (a-f.i), show 3D views; bottom row, panels (a-f.ii), show 2D views.

AFM Details of the PDMS Metasurface Showing Nanocones

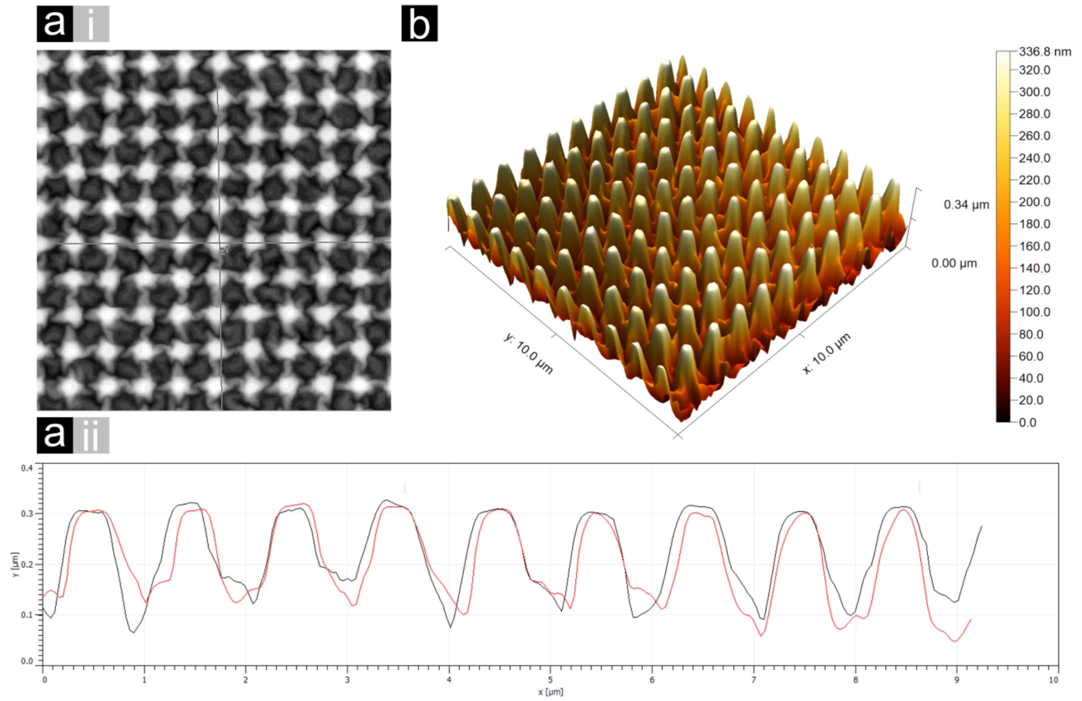

Figure S3. (a-i) 2D AFM height map of the NC-TENG with 1000 nm periodicity. (a-ii) Corresponding line profiles taken along the x (black) and y (red) axes showing uniform periodicity. (b) 3D AFM height map used to render Fig. 1b in the main text.

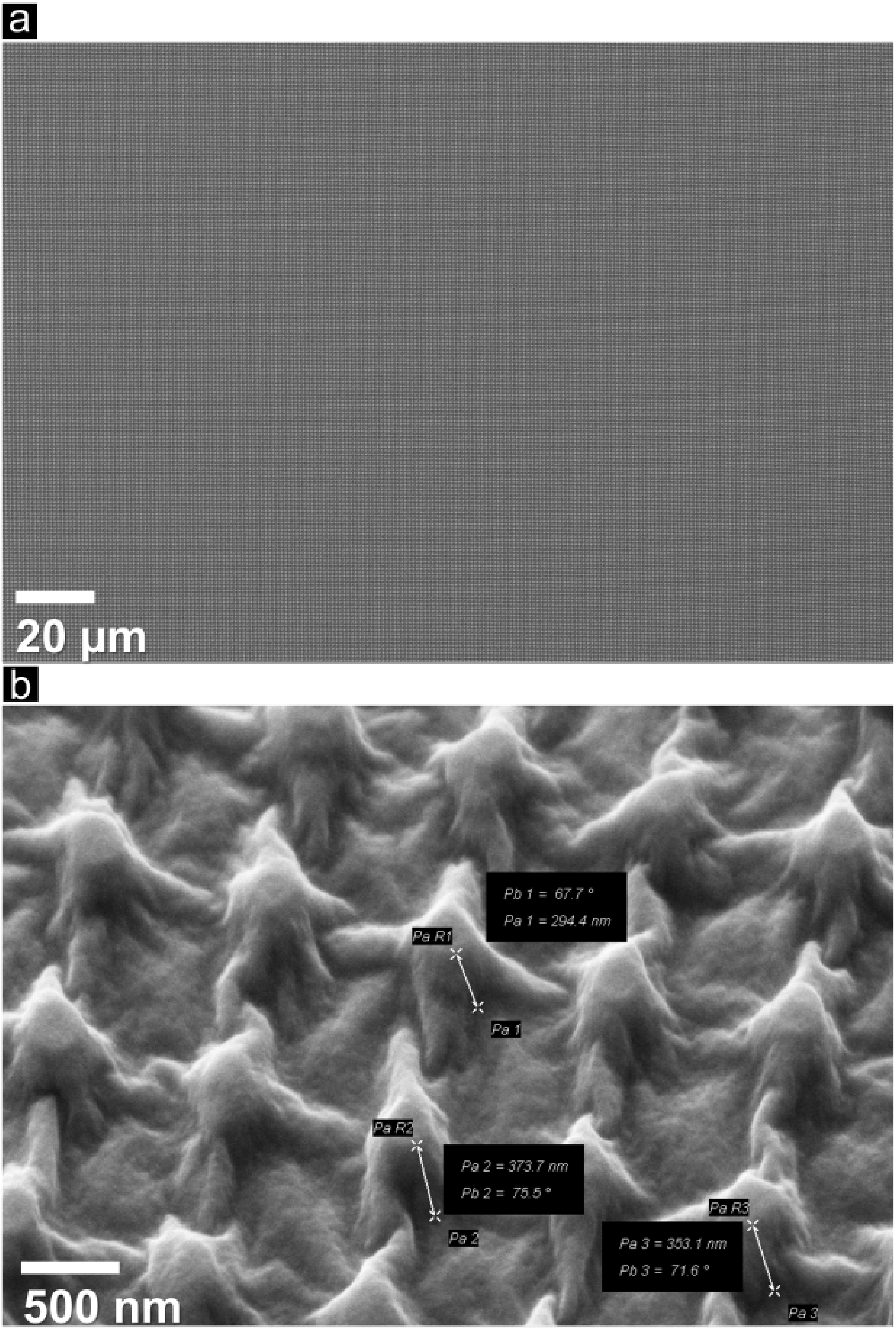

Figure S4. (a) Low-magnification SEM image showing a uniform large-area distribution of LIL-based PDMS nanocones. (b) High-magnification SEM revealing the detailed nanocone geometry, including periodic cone-to-cone spacing (~300 nm–380 nm) and apex angles (~68°–76°), confirming well-defined nanocone geometry responsible for enhanced triboelectric contact area and local electric-field concentration in the NC-TENG.

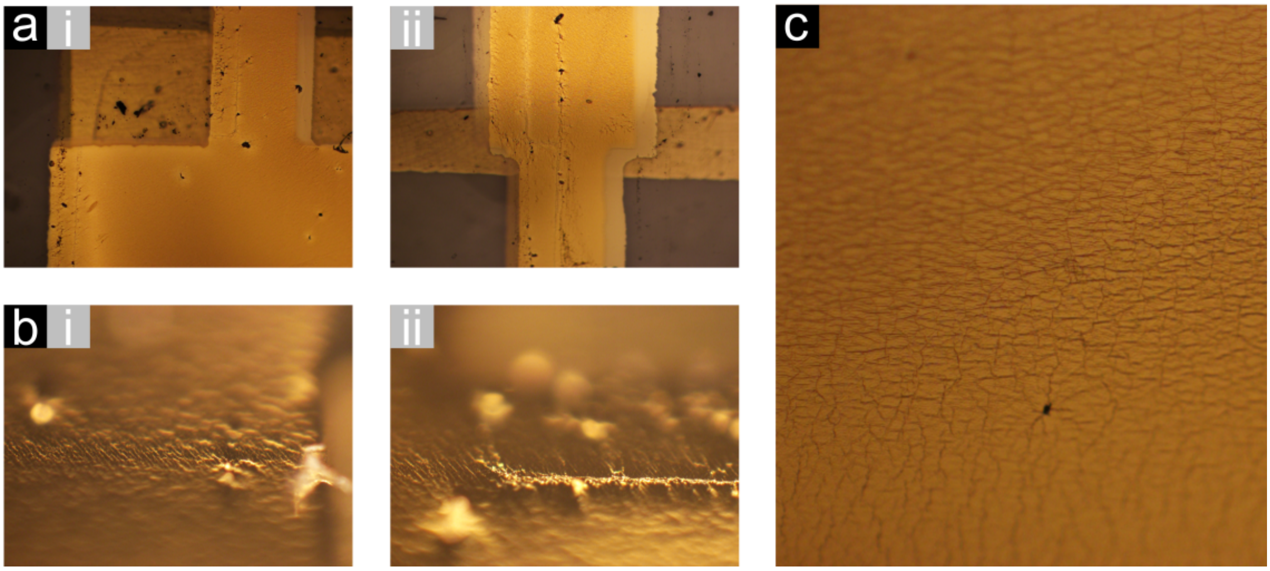

Figure S5. Optical micrographs of the NC-TENG electrodes and connectors. (a.i-ii) Top-view images at 5× magnification. (b.i-ii) Tilted-view images at 20× magnification. (c) Surface relief of an electrode at 20× magnification.

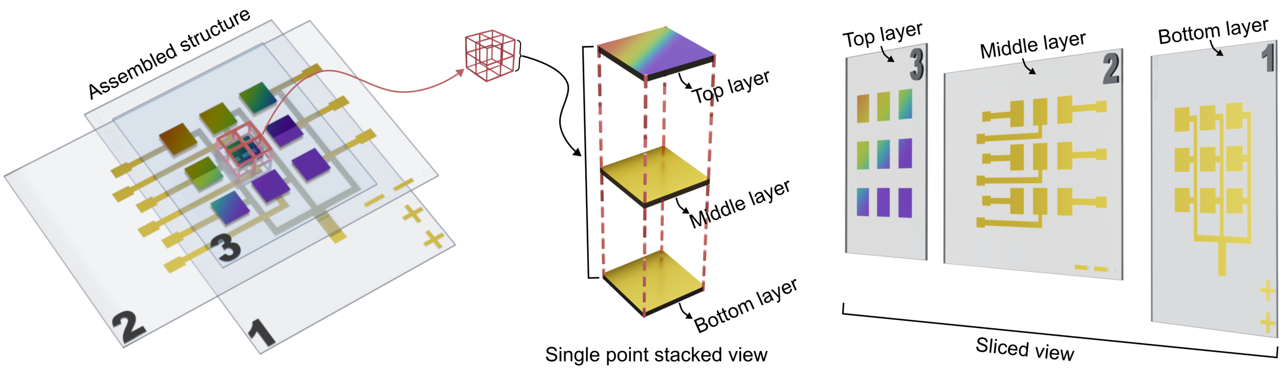

Figure S6. Schematic of the trilayer NC-TENG device architecture. The assembled structure (left), single-point stacked view (center), and sliced view of the three PDMS-based layers (right; bottom electrode = 1, top electrode = 2, patterned PDMS metasurface = 3) illustrate the vertical stacking and lateral alignment of the metasurface pixels with the underlying stretchable electrodes.

The left sub-figure shows an oblique view of the fully assembled structure, consisting of three PDMS-based layers: the bottom electrode layer (1), the top electrode layer (2), and the patterned PDMS metasurface layer (3). The coloured squares correspond to individual metasurface pixels that serve as active TENG units.

The central sub-figure ("single-point stacked view") magnifies one representative pixel region and depicts the vertical stacking of the three layers, highlighting how the metasurface pixel on the top PDMS layer (3) is aligned with the corresponding electrode pads on the middle (2) and bottom (1) layers.

The right sub-figure ("sliced view") presents the same structure with the three layers separated for clarity. From right to left, the bottom electrode layout (layer 1), the top electrode layout (layer 2), and the patterned PDMS metasurface (layer 3) are shown. The numerical labels 1-3 are used consistently from bottom to top to help the reader visualize the relative position and registration of each layer in the final assembled device.

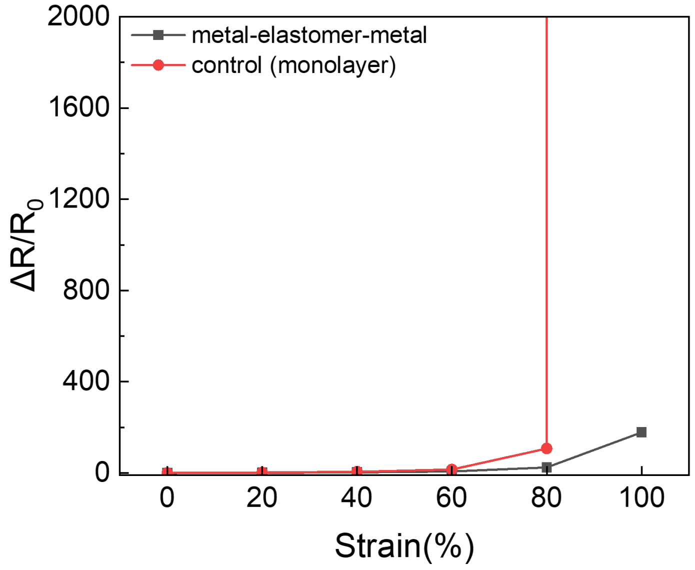

Figure S7. Relative resistance change ($\Delta R/R_0$) versus applied tensile strain rate for a metal-elastomer-metal sandwich nanostructure compared with a monolayer metal thin film.

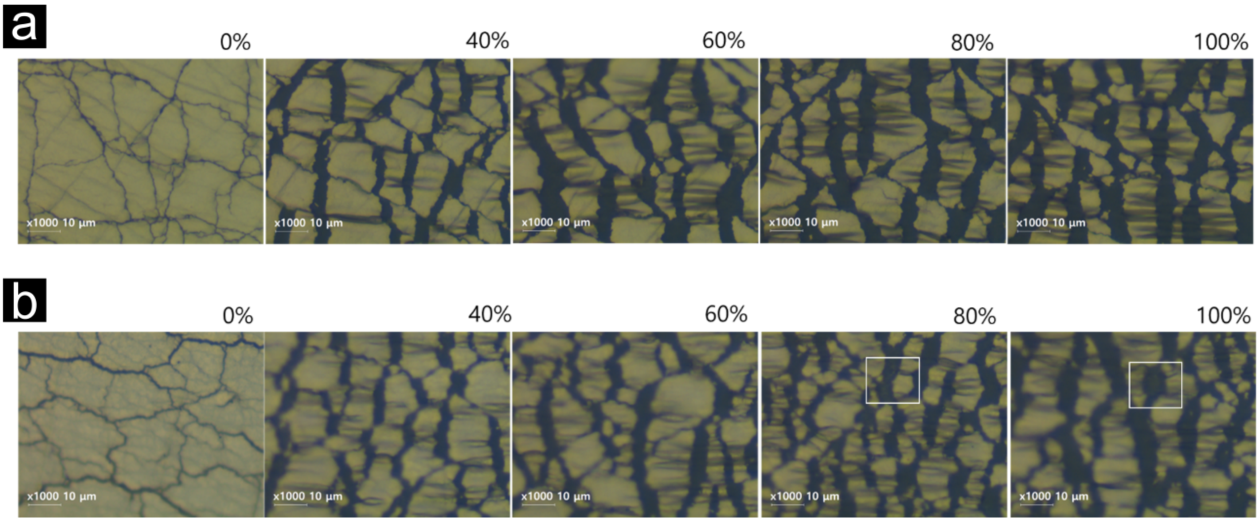

Figure S8. Optical micrographs of the surface morphology of (a) the control monolayer metal thin film and (b) the metal-elastomer-metal (sandwich) structure under varying tensile strain. Unlike the monolayer, the sandwich nanostructure shows exposed underlying Au within crack regions, highlighted by white rectangles.

Table S1. Volume resistance and conductivity measurement data.

Resistance [Ω]	Distance electrodes [cm]	thickness [nm]	thickness [cm]	Pin width [mm]	Pin width [cm]	Area [cm^2]	Vol. resistance [Ω·cm]	Vol. resistance [Ω·m]	Conductivity [S/m]
300	0.3	50	5E-6	0.2	0.02	1.00E-7	1.00E-4	1.00E-06	1.00E+06
600	0.5						1.20E-4	1.20E-06	8.33E+05
600	1.2						5.00E-5	5.00E-07	2.00E+06

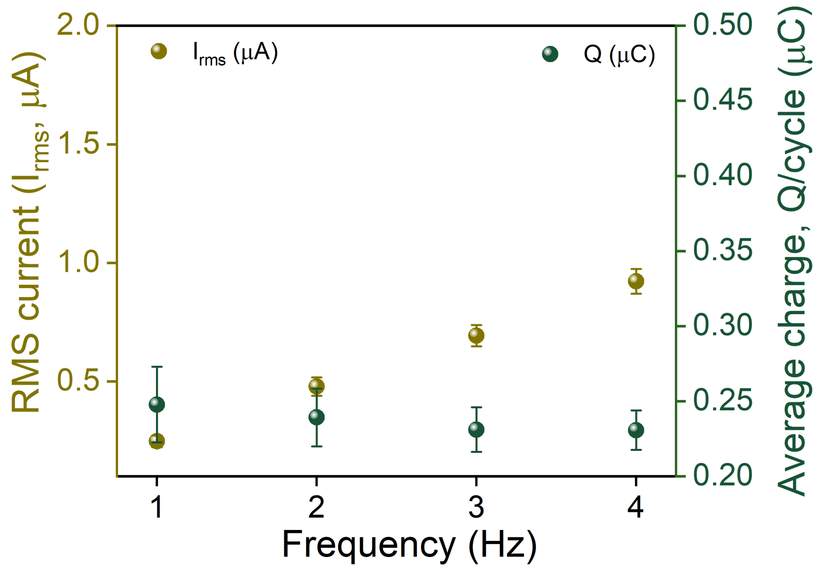

Figure S9. RMS current and average transferred charge with respect to frequency for NC-TENG.

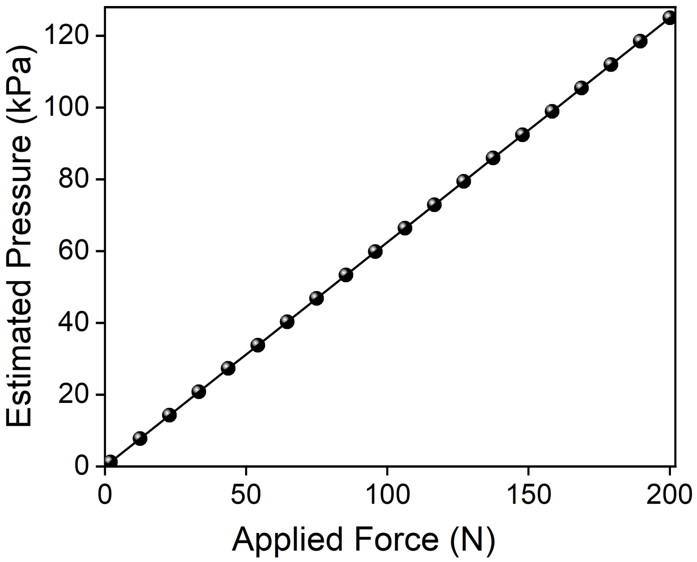

Figure S10. Calibration chart for converting normal force (N) into nominal contact pressure (kPa).

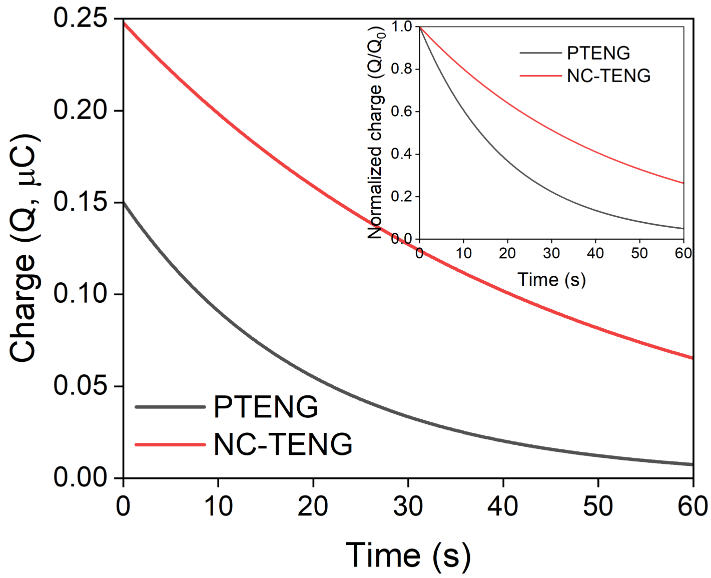

Figure S11. Charge-dissipation plots including absolute charge decay curves Q(t) for NC-TENG and PTENG, and normalized decay curves $Q(t)/Q_0$.

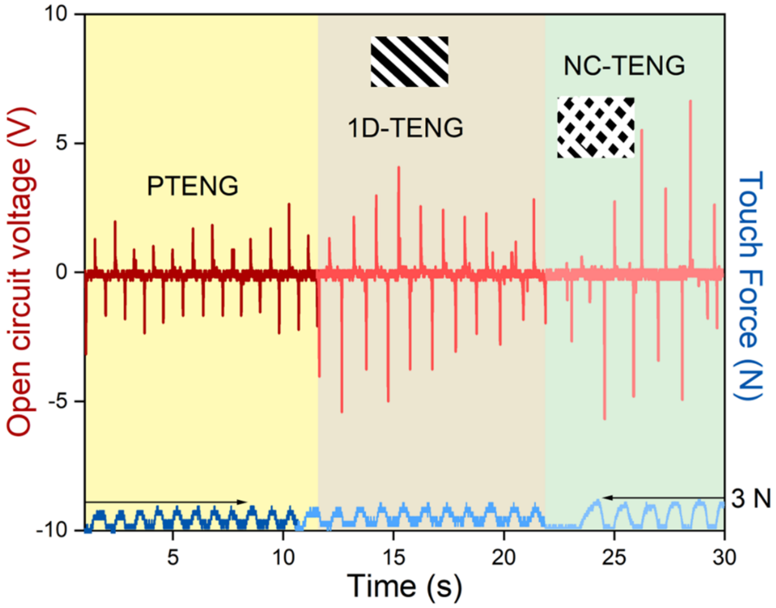

Figure S12. Open-circuit voltage of three TENG architectures under identical finger-touch conditions (~ 3 N). The untextured PTENG shows the lowest signals. The 1D-patterned TENG yields higher output due to enhanced contact electrification. The NC-TENG produces the largest and most consistent waveforms, indicating superior charge generation from the nanocone surface.

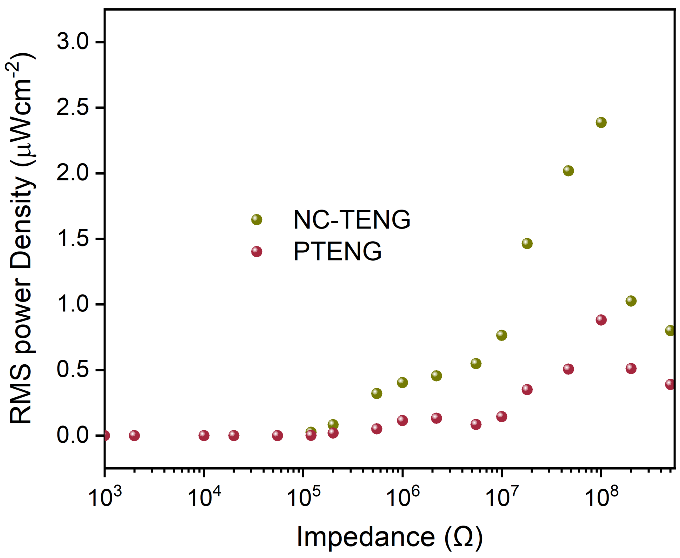

Figure S13. RMS power density as a function of load impedance for NC-TENG and PTENG.

Long-Term Ambient Stability and Effect of Humidity on Electrical Output

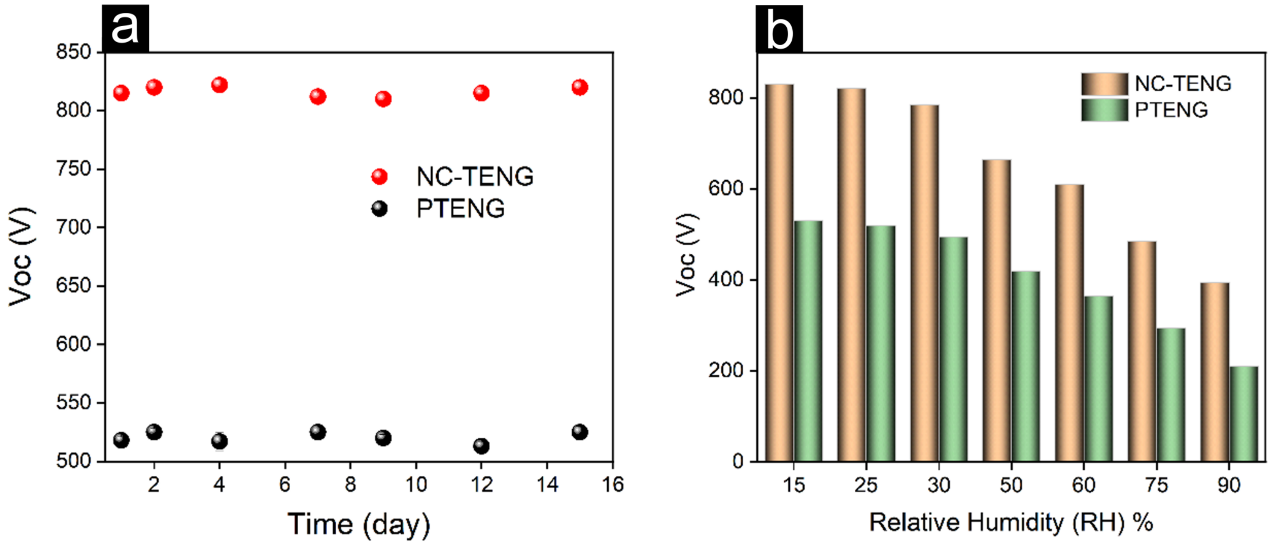

Figure S14. (a) Long-term ambient stability of NC-TENG and PTENG over 15 d. NC-TENG maintains V_{OC} (> 800 V) with negligible degradation, while PTENG remains lower at ~520 V yet similarly stable, indicating no charge decay under normal storage. (b) Effect of relative humidity (RH) on output. Both show a monotonic decrease in V_{OC} as RH increases from 15 % to 90 %, consistent with moisture-induced surface-charge screening. NC-TENG retains higher output than PTENG at all humidity levels, confirming the metasurface benefit for maintaining effective charge density.

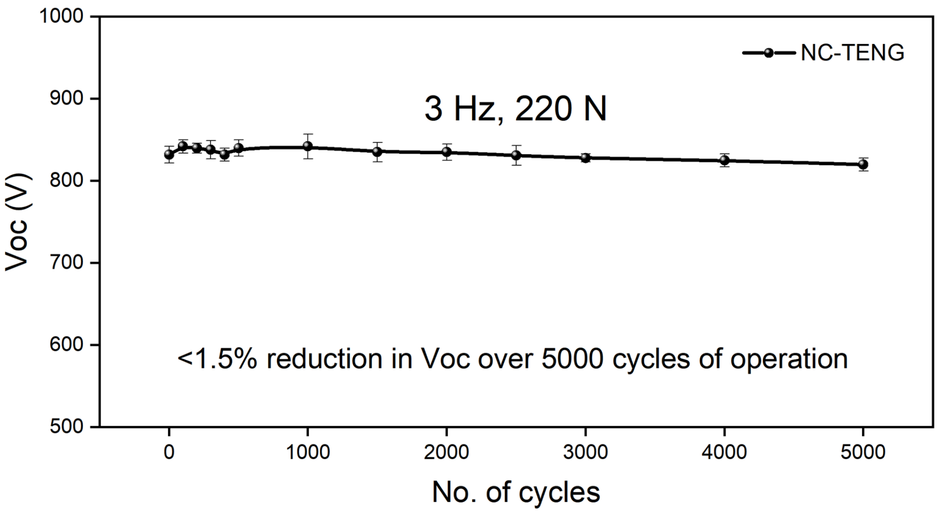

Figure S15. Long-term durability of the NC-TENG over 5000 continuous contact-separation cycles at 3 Hz and an approximately 220 N normal force.

Strain-Dependence of V_{OC} in NC-TENG and PTENG

1. thickness: $d = \frac{d_0}{\lambda}$
2. area: $A = \lambda A_0$
3. total charge approximately conserved:

$$Q = \sigma_0 A_0 = \sigma_{eff} A$$

Therefore,

$$\sigma_{eff} = \frac{Q}{A} = \frac{\sigma_0}{\lambda}.$$

Inserting into the expression of V_{OC},

$$V_{OC}(\lambda) = \frac{\sigma_{eff}\, d}{\varepsilon_0 \varepsilon_r} = \frac{\sigma_0/\lambda}{\varepsilon_0 \varepsilon_r} \cdot \frac{d_0}{\lambda} = V_{OC}(0) \cdot \frac{1}{\lambda^2},$$

where $V_{OC}(0) = \frac{\sigma_0 d_0}{\varepsilon_0 \varepsilon_r}$.
Finally,

$$V_{OC} \propto \lambda^{-2}.$$

Therefore, the open-circuit voltage declines quadratically with tensile stretch because charge density per area decreases and capacitance increases as the dielectric layer is thinned under stress.

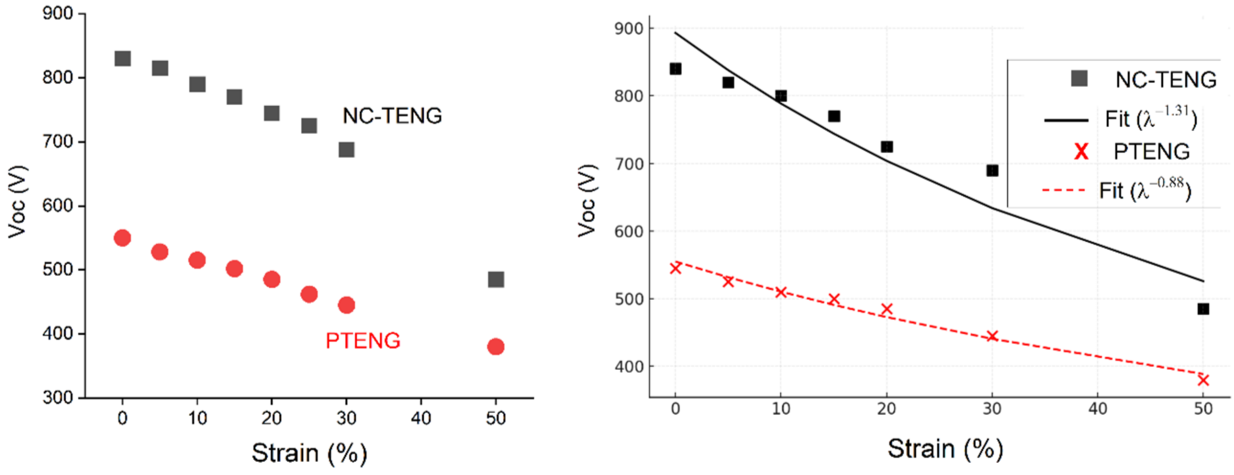

Figure S16. Strain-dependent open-circuit voltage of NC-TENG and PTENG. NC-TENG delivers higher V_{OC} for all applied strains from 0 %–50 %, confirming enhanced charge generation due to the nanocone surface topography.

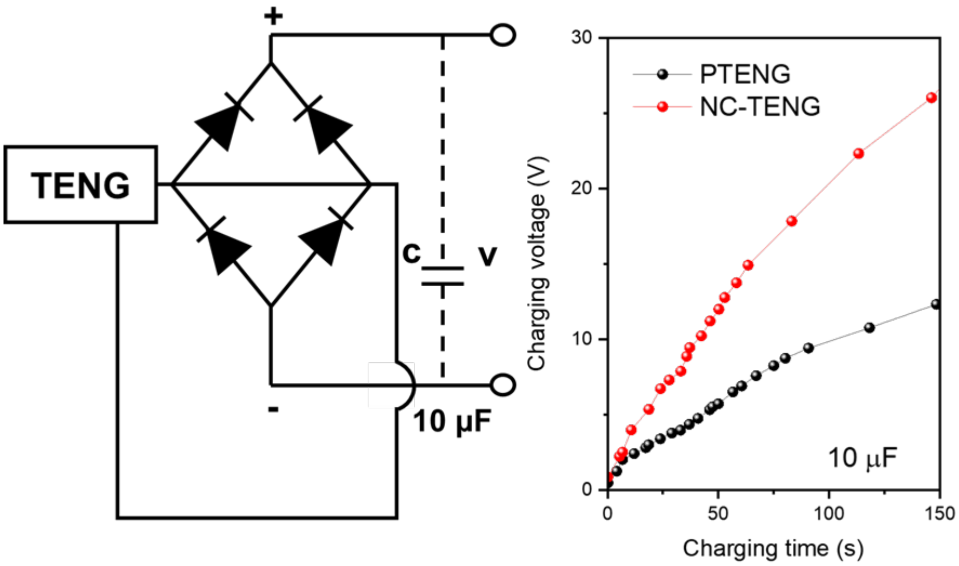

Figure S17. Circuit diagram for charging a 10 μF capacitor using a bridge rectifier (left). Charging curves for the 10 μF capacitor with NC-TENG and PTENG (right).

Table S2. TENG response characteristics of textured and flat PDMS surfaces in contact with a flat copper surface.

Surface type	Open-circuit voltage (V)	Short-circuit charge density ($C\cdot mm^{-2}$)	Maximum power density ($mW\cdot cm^{-2}$)	Time-averaged power density ($mW\cdot cm^{-2}$)
Smooth	10.4	2.43×10^{-11}	1.1	0.4
Textured	19.6	3.47×10^{-11}	2.9	1.0
Textured-Homogenized	15.2	3.56×10^{-11}	2.4	0.8

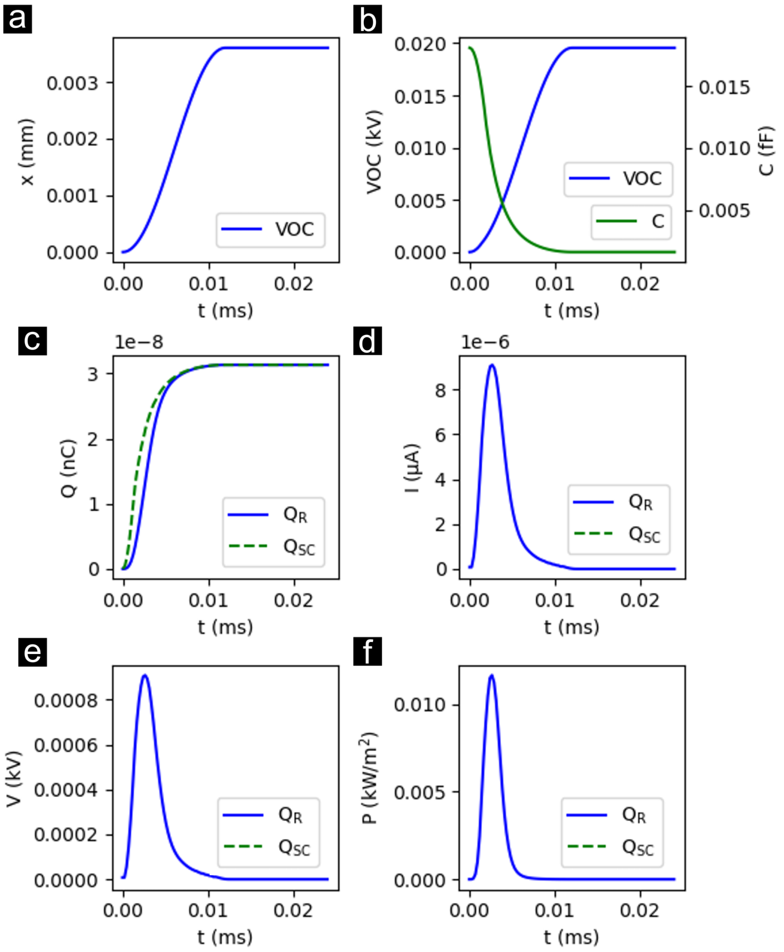

Figure S18. Time-domain responses for a textured-PDMS/smooth-Cu TENG with an external load of 1011 Ω: (a) displacement, (b) internal capacitance and open-circuit voltage, (c) transferred charge, (d) current, (e) voltage, and (f) power.

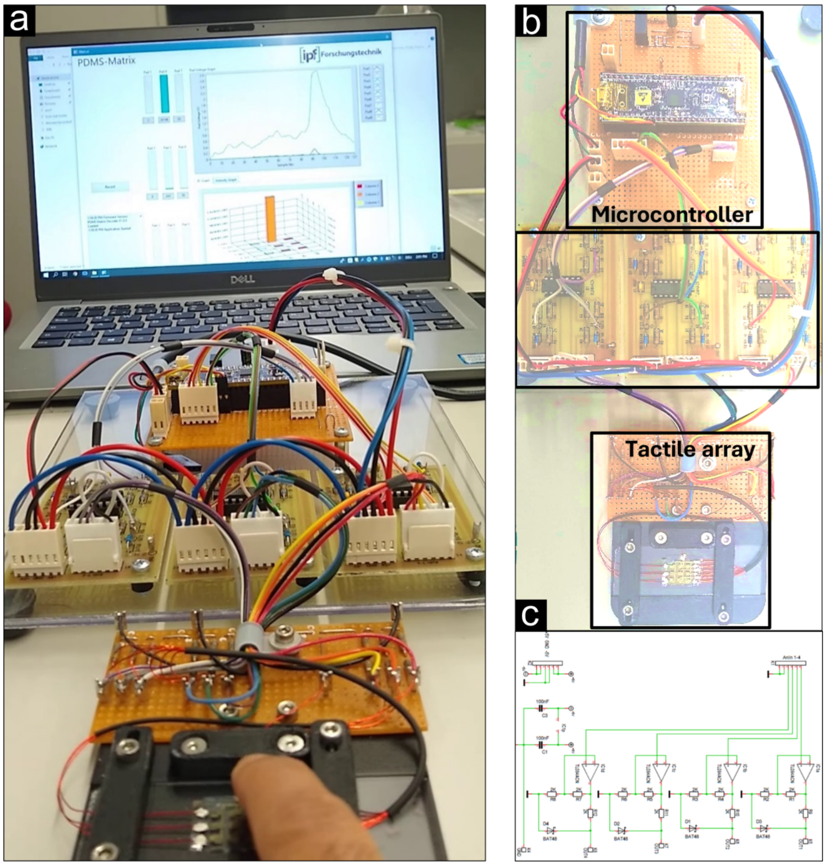

Figure S19. (a) Experimental setup of the tactile triboelectric active matrix (TTAM) interfaced with a laptop for real-time data acquisition and visualization. (b) Electronic architecture of the TTAM showing the integrated microcontroller for multiplexed signal routing and amplification. (c) Circuit schematic of the TTAM readout, including amplification and signal-conditioning stages.

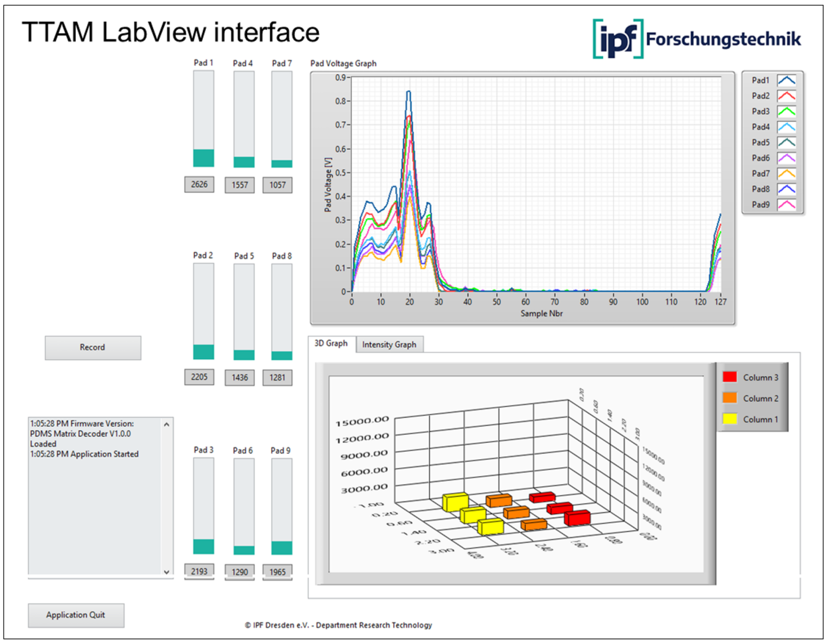

Figure S20. Custom LabVIEW interface for the TTAM developed at IPF Dresden. The software enables real-time visualization and logging of triboelectric signals from individual pads: pad-wise voltage monitoring (top), time-resolved waveform acquisition (center), and spatial mapping of touch intensity with a 3D view (bottom). The interface tracks both tactile and non-tactile responses for active-matrix operation and interaction analysis.

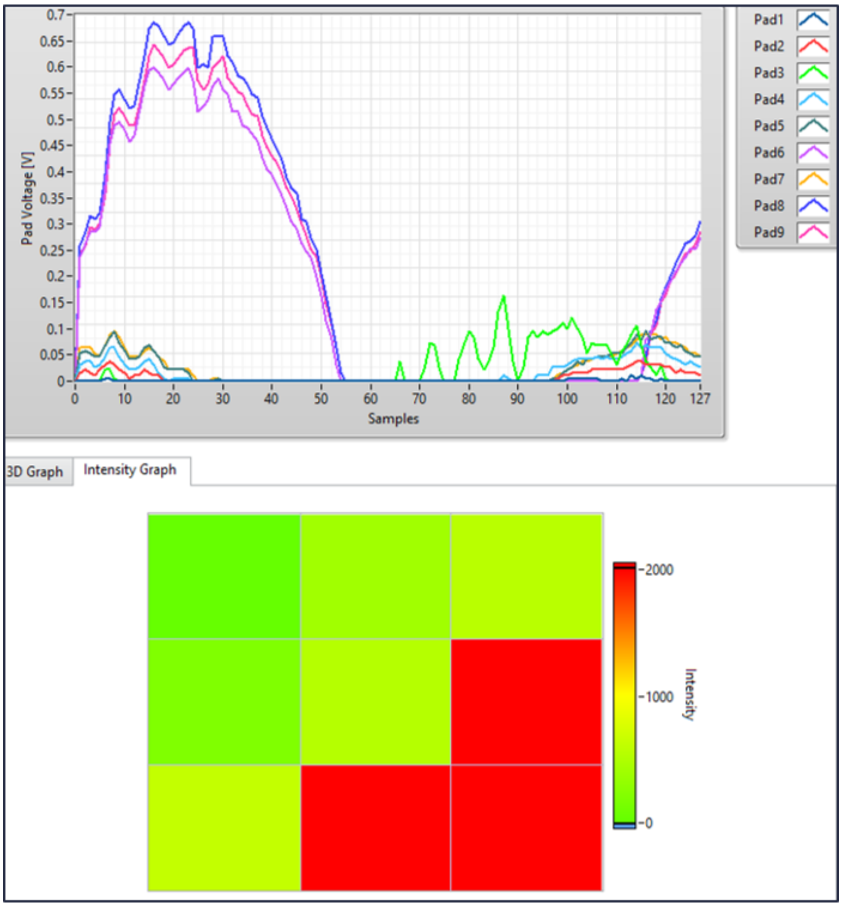

Figure S21. Proximity-sensing performance of the TTAM. Top: Voltage response of a nine-pad array when a finger hovers near the corner of Pad 9 without contact; only nearby pads show induced signals due to capacitive coupling to the tribo-charged metasurface. Bottom: Corresponding 2D intensity map showing a localized rise at Pad 9, while other pads remain near baseline.

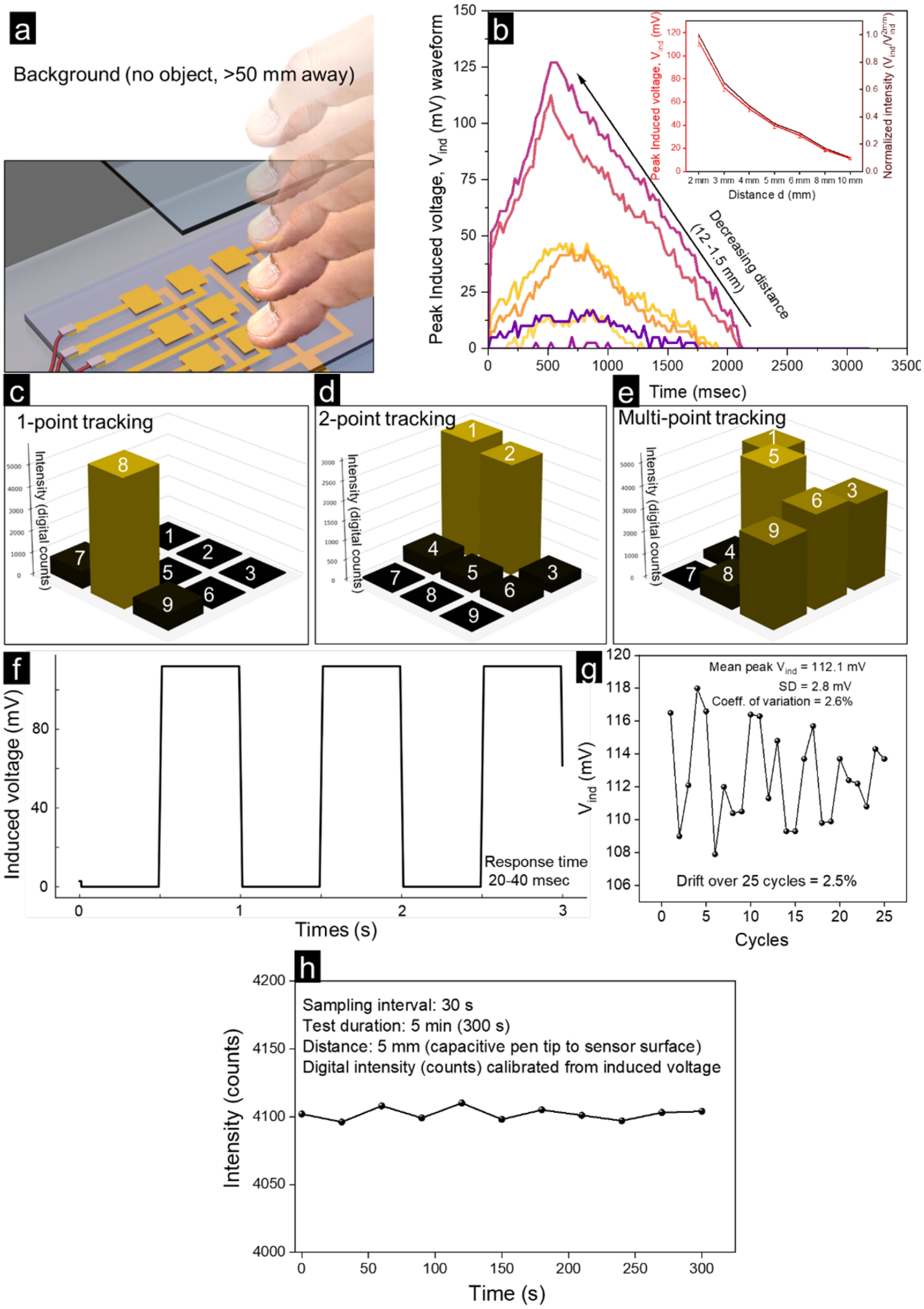

Figure S22. Non-contact proximity sensing with the nanocone-PDMS metasurface. (a) Schematic of the non-contact sensing mode with a grounded human finger. (b) Distance-dependent induced voltage V_{ind} for multiple separations. (c-h) Proximity tracking and stability tests on a 3×3 pixel array.

The working principle of the non-contact sensing mode is illustrated in Fig. S22a. A grounded human finger placed a few millimetres (2–10 mm) above the nanocone-PDMS metasurface perturbs the local triboelectric potential in the near field, inducing a measurable voltage on the underlying electrode. The distance dependence of this effect is quantified in Fig. S22b, which shows the induced voltage V_{ind} recorded at several finger-surface separations. As the separation increases, the induced voltage decreases monotonically, consistent with the reduced capacitive coupling between the finger and the metasurface. The inset reports the corresponding normalized intensity, highlighting the gradual decay of the signal with distance and defining the effective non-contact sensing range.

The spatially resolved proximity sensing performance on the 3×3 pixel array is summarized in Fig. S22c-h. A single-point proximity event is visualized in Fig. S22c, where only pixel 8 is activated, demonstrating localized addressability of an individual pad. Two-point proximity tracking is shown in Fig. S22d, where pads 1 and 2 are simultaneously activated, illustrating the ability to resolve multiple non-contact targets in space. Figure S22e presents multi-point proximity tracking with sequential activation of pads 1, 5, 9, 6, and 3, demonstrating that the array can map and track the motion of a non-contact stimulus across the surface. The time-resolved response in Fig. S22f captures repeated approach-hold-withdraw cycles, from which a characteristic response time of approximately 20–40 ms is extracted. The corresponding spatial activation pattern over 25 repeated cycles is shown in Fig. S22g, confirming robust and repeatable localization. Finally, Fig. S22h shows the signal stability monitored at a single pixel over a 5 min interval, indicating that the proximity response remains stable over extended operation without noticeable drift.

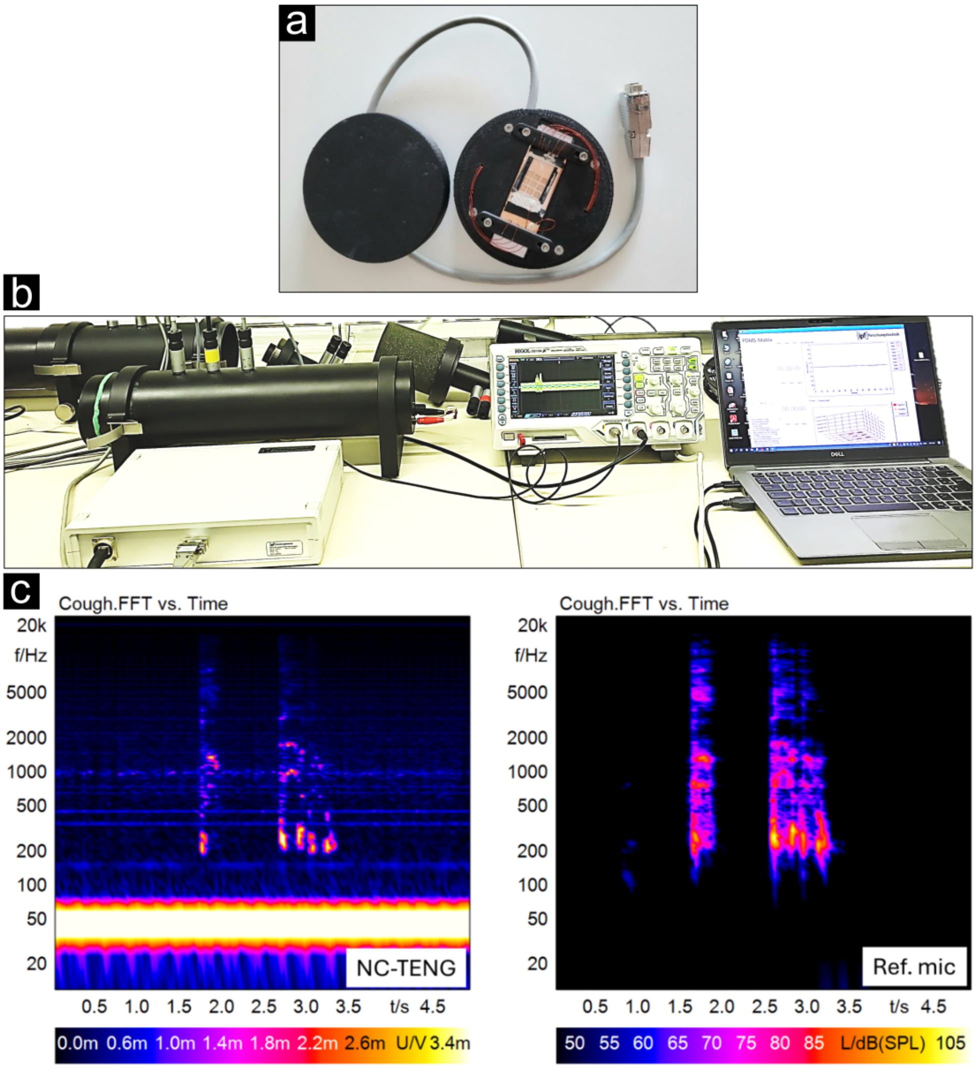

Figure S23. (a) NC-TENG acoustic-sensing module with integrated nanocone-enabled microphone patch and signal cable. (b) Measurement setup for acoustic sensing with the NC-TENG sensor, a reference microphone, a digital oscilloscope, and a data-acquisition system. (c) Time-frequency spectrograms (STFT) of a human cough recorded by the NC-TENG (left) and a calibrated reference microphone (right). The NC-TENG resolves the broadband spectral signatures of the cough, validating self-powered acoustic sensing.

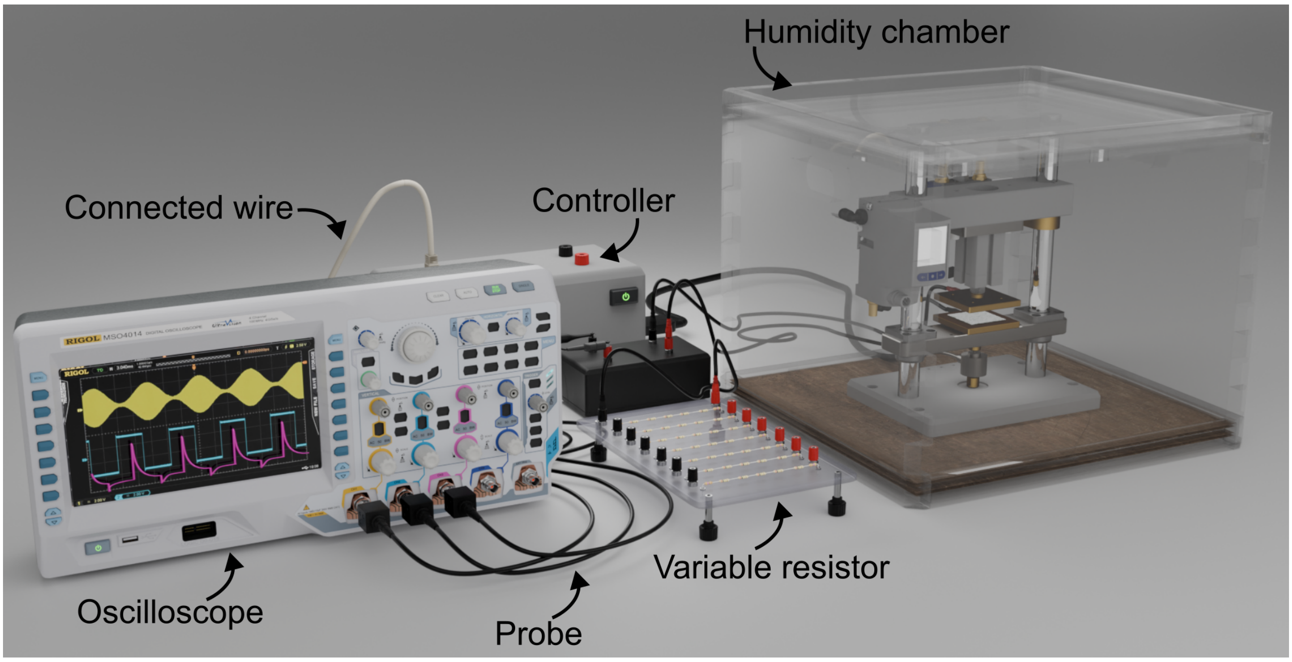

Figure S24. In-house developed triboelectric test setup with multichannel DAQ, humidity chamber, force sensor, and controller connected.

The output characteristics of the NC-TENG (and reference PTENG) were measured using the vertical contact-separation test setup shown in Figure S24. The device was mounted on a pneumatic actuator (Festo) inside a closed humidity chamber, enabling testing under controlled environmental conditions. The actuator can deliver normal forces up to 500 ± 20 N; for the measurements in Fig. 3b-f the contact force was fixed at 200 ± 20 N, corresponding to 3 bar, while the bottom plate remained stationary and the top plate was driven in vertical contact-separation. The actuation frequency and contact time were set using a custom controller. The electrical output of the NC-TENG was routed outside the chamber via shielded connection wires to a variable-resistor board providing discrete load resistances, and then to a three-channel oscilloscope (Rigol DS4024), where channel 1 monitored the trigger signal, channel 2 recorded the open-circuit voltage V_{oc}, and channel 3 measured the contact force via an in-line load cell. All electrical and mechanical signals were synchronously acquired using a National Instruments NI-9239 data-acquisition module (USB carrier) and a LabVIEW interface, enabling time-resolved recording of V_{oc}, I_{sc}, transferred charge Q, and the corresponding actuation parameters.

Table S3. Comparative table with key benchmark metrics demonstrating current state-of-the-art on triboelectric acoustic sensors (TAS).

**Published paper**	**Device/type**	**Frequency (Hz)**	**SPL range (dB)**	**Sensitivity (V/Pa; dB re 1 V/Pa)**	**SNR (dB)**
**This work: NC-TENG acoustic patch**	NC-TENG metasurface acoustic patch (nanocone elastomer membrane on back-cavity substrate; triboelectric PDMS/Au)	50–6400 (multi-resonant)	≈ 60–112 (speaker-limited)	5.3 mV Pa^{-1} (0.0053 V Pa^{-1}) ≈ 45.6 dB	≈ 33 dB during speech segments; ≈ 24 dB over 10 s
Sun et al.[1]	TTAS (tiny triboelectric acoustic sensor) based on NFM-TENG with PVDF-MWCNTs (1 wt%) nanofibrous membrane (tribo-positive) and corona-charged FEP membrane (tribo-negative)	20–20000 Hz	Calibrated per IEC61094 (0 dB = 1 V Pa^{-1})	Calibrated sensitivity −50 dB re 1 V Pa^{-1} (≈ 3.2 mV Pa^{-1})	Not reported
Hui et al.[2]	Acoustically enhanced triboelectric stethoscope: FEP triboelectric membrane with Al and PI membrane sputtered by Au layer, combined with a trumpet-shaped auscultatory cavity for acoustic energy converging and impedance matching	Cardiac band 20–600 Hz (low-frequency heart sounds)	50–80 dB SPL (cardiac test conditions)	Ultrahigh sensitivity 1215 mV Pa^{-1} (1.215 V Pa^{-1} ≈ +1.7 dB re 1 V Pa^{-1}); for comparison, piezoelectric counterpart ~ 21 mV Pa^{-1}	Signal-to-noise ratio ≈ 36 dB in human test (~ 2.3× higher than piezoelectric stethoscope)
Yu et al.[3]	Underwater low-frequency TENG hydrophone (PTFE/Cu + PET/Cu tribo-pair inside sealed cubic cavity)	30–200 Hz (resonant at 110 Hz)	Acoustic pressure ≈ 133–155 dB SPL; 144.2 dB SPL at resonance	Sensitivity > −185 dB over 30–200 Hz; peak sensitivity −146 dB at 110 Hz (dB re 1 V/μPa, hydrophone convention)	Not reported
Shao et al.[4]	Triboelectric AI acoustic textile (A-Textile); SnS_2 nanoflower-doped silicone rubber charge-capture/transport layer + SnS_2 NFs-decorated carbonized textile charge reservoir	80–900 Hz (voice band; 1 Hz resolution)	70–120 dB SPL	Sensitivity up to 1.2 V Pa^{-1} at 80 dB SPL (≈ +1.6 dB re 1 V Pa^{-1})	SNR 59.3 dB; 24.4 dB at 70 dB SPL, 35.8 dB at 80 dB SPL
Yang et al.[5]	DS-TAS (dual-symmetry triboelectric acoustic sensor; triboelectric diaphragm/cavity architecture)	20 Hz–200 kHz (audible + ultrasonic)	0.002–0.02 Pa (≈ 40–60 dB); calibrated up to ~ 92 dB at 1 kHz	Reported sensitivity 6.67 V Pa^{-1} (≈ +16.5 dB re 1 V Pa^{-1})	Not reported

Published paper	Device/type	Frequency (Hz)	SPL range (dB)	Sensitivity (V/Pa; dB re 1 V/Pa)	SNR (dB)
Li et al.[6]	FENG-based dual-functional loudspeaker/microphone (ferroelectret PP foam with silicate-induced voids + Ag electrodes)	10–20000 Hz (SPL response characterized for loudspeaker; microphone demonstrated over human-audible band)	≈ 60–110 dB SPL depending on area/layer number; ~ 73 dB SPL when laminated on acrylic substrate	Microphone sensitivity not given numerically; high electromechanical coupling enables high-fidelity recording of music/voice	High-fidelity sound reproduction and clear music/voice recording (qualitative SNR; no explicit value reported)
Guo et al.[7]	TAS (triboelectric auditory sensor for social robotics and hearing aids; circular membrane with conductive top electrode and tribomaterial bottom layer)	100–5000 Hz (broad human-voice range)	Speech-range SPL, typically 85–110 dB SPL	Ultrahigh sensitivity 110 $mV\,dB^{-1}$; reported sensitivity ~ 513.9 $mV\,Pa^{-1}$ over 85–110 dB SPL	Not explicitly quantified (high-quality music recording and accurate voice recognition demonstrated)

Table S4. Mechanical properties of the silicone (PDMS) substrate used for TENG fabrication (1 mm dog-bone specimen).

Sample	Tensile strength (MPa)	Young's modulus (MPa)	Elongation at break (%)
PDMS (as per ISO 37/ASTM D412)	1.8 ± 0.11	1.3 ± 0.15	118.4 ± 5

References

[1] W. Sun, J. Chen, T. Yuan, D. Sui, J. Zhou, *Nano Energy* **2024**, *128* 109913.

[2] X. Hui, L. Tang, D. Zhang, S. Yan, D. Li, J. Chen, F. Wu, Z. L. Wang, H. Guo, *Advanced Materials* **2024**, *36*, 29 2401508.

[3] A. Yu, M. Song, Y. Zhang, Y. Zhang, L. Chen, J. Zhai, Z. L. Wang, *Nano Research* **2015**, *8*, 3 765.

[4] B. Shao, T.-C. Wu, Z.-X. Yan, T.-Y. Ko, W.-C. Peng, D.-J. Jhan, Y.-H. Chang, J.-W. Fong, M.-H. Lu, W.-C. Yang, J.-Y. Chen, M.-Y. Lu, B. Sun, H.-J. Liu, R. Liu, Y.-C. Lai, *Science Advances* **2025**, *11*, 41 eadx3348.

[5] H. Yang, X. Xiao, F. Manshaii, D. Ren, X. Li, J. Yin, Q. Li, X. Zhang, S. Xiong, Y. Xi, J. Chen, *Nano Energy* **2024**, *126* 109638.

[6] W. Li, D. Torres, R. Díaz, Z. Wang, C. Wu, C. Wang, Z. Lin Wang, N. Sepúlveda, *Nature Communications* **2017**, *8*, 1 15310.

[7] H. Guo, X. Pu, J. Chen, Y. Meng, M.-H. Yeh, G. Liu, Q. Tang, B. Chen, D. Liu, S. Qi, C. Wu, C. Hu, J. Wang, Z. L. Wang, *Science Robotics* **2018**, *3*, 20 eaat2516.
